# Siglec-E augments adipose tissue inflammation by modulating TRAF3 signaling and monocytic myeloid-derived suppressor cells during obesity

**DOI:** 10.3389/fimmu.2025.1501307

**Published:** 2025-02-04

**Authors:** Ahmed Rakib, Mousumi Mandal, Md Abdullah Al Mamun, Sonia Kiran, Nelufar Yasmen, Lexiao Li, Daniel M. Collier, Jianxiong Jiang, Frank Park, Udai P. Singh

**Affiliations:** Department of Pharmaceutical Sciences, College of Pharmacy, University of Tennessee Health Science Center, Memphis, TN, United States

**Keywords:** adipose tissue, CXCR3, MDSCs, siglec-E, TRAF3

## Abstract

**Background:**

Obesity is associated with dysregulated metabolism and low-grade chronic inflammation in adipose tissue (AT). Immune cells, including macrophages, T cells, and neutrophils, infiltrate the AT and secrete proinflammatory cytokines to exacerbate the AT inflammation. RNA-Seq analysis of AT immune cells isolated from mice fed a high-fat diet (HFD) versus normal fat diet (ND) identified a panel of genes that were markedly downregulated, including sialic acid-binding Ig-like lectin E (siglec-E), in HFD compared to ND mice.

**Methods:**

A series of experiments in wild-type (WT) and siglec-E knockout (siglec-E KO) mice was designed to investigate the effect of HFD on the functional role of siglec-E in the regulation of AT inflammation and adipogenesis. We analyzed the changes in immune phenotypes, inflammatory response, adipogenesis, and levels of cytokines and chemokines after HFD and ND feeding.

**Results:**

HFD consumption significantly increased the body weight and blood glucose levels in siglec-E KO mice relative to those of WT mice. This was associated with an increased infiltration of macrophages, CXCR3 expressing CD8 T cells, and monocytic myeloid-derived suppressor cells (M-MDSCs) with a concomitant decrease in numbers of dendritic cells (DCs), in the AT of siglec-E KO fed HFD versus the WT HFD counterparts. The HFD-fed siglec-E KO mice also exhibited elevated expression of intracellular Akt and TNF receptor-associated factor 3 (TRAF3) signaling, inducing C/EBPα, FASN, PPARγ, and resistin in suprascapular AT compared to WT HFD-fed mice. Taken together, these results suggest that a genetic deficiency of siglec-E plays a key role in inducing AT inflammation by differentially altering M-MDSCs and CD8^+^CXCR3^+^ T cell function and adipogenesis by TRAF3 and Akt signaling in AT.

**Conclusion:**

Our findings strongly suggest that modulation of siglec-E pathways might have a protective effect at least in part against AT inflammation and metabolic disorders.

## Graphical Abstract

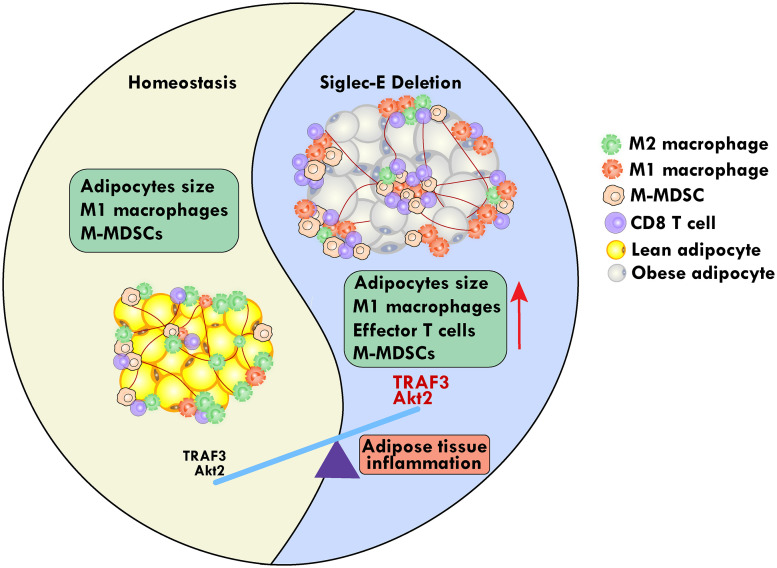

## Introduction

The obesity epidemic spread across the world and is characterized by abnormal or excessive accumulation of fat that exhibits a health risk to the entire globe (1). Obesity is responsible for the development of several metabolic abnormalities including, cardiovascular disease, type 2 diabetes mellitus (T2DM), metabolic syndrome, and various immune-mediated disorders (2–4). Further, obesity is also associated with adipocyte hypertrophy and hyperplasia, which facilitates the storage of lipid bodies, and increased immune cell infiltration in the adipose tissue (AT). The predominant form of whole-body AT is white AT (WAT), which stores energy as triglycerides, and its increased accumulation contributes to obesity and several other metabolic disorders (5). In contrast, brown AT contains several small lipid droplets and an increased number of mitochondria for energy expenditure and plays a role in adaptive thermogenesis. During obesity, adipocyte triglycerides undergo enhanced hydrolysis to increase circulating free fatty acids levels and induce the development of insulin resistance and other metabolic disorders (6). Thus, resetting the key signaling in AT during obesity, which maintains low-grade chronic inflammation will provide an innovative approach to the management and therapeutics of obesity.

In addition to adipocytes, AT infiltrates various immune cells, including macrophages, neutrophils, natural killer (NK) cells, and T cells that exhibit distinct phenotypes in healthy and obese conditions (7). The chemokine receptor CXCR3 and its ligands facilitate immune cell trafficking in tissue and CXCR3-expressing adipocytes modulate inflammation during obesity (8). Further, CXCR3 expressing CD8^+^ T cells also play a crucial role in diet-induced obesity (9). The NKT cells initiate obesity-related inflammation and insulin resistance (10), in contrast, AT-invariant NKT cells produce regulatory cytokines, which protect mice from HFD-induced obesity (11). AT-resident macrophages synergistically coordinate with adipocytes to orchestrate the body’s energy metabolism (12). During energy stimulation, AT alters the expansion of adipocytes and macrophages to a proinflammatory phenotype and recruits proinflammatory mediators, including interleukin (IL)-1β and tumor necrosis factor (TNF)-α (13). The heterogeneous myeloid cell population called myeloid-derived suppressor cells (MDSCs), mediate both adaptive and innate immune responses during inflammation. Two types of MDSCs, monocytic and granulocytic MDSCs (M-MDSCs and G-MDSCs) mediate T cell anergy, suppress T cell activation, induce inflammation, and inhibit the cytotoxicity of NK cells (14). Adipocytes secrete adipokines may trigger the function of MDSCs. Further, adipogenesis is promoted by the Akt signaling pathway in which Akt2 maintains AT (15) and the downstream mammalian target of rapamycin (mTOR) facilitates MDSCs functions (16). It has been shown that during the early stages of obesity, MDSCs accumulate in the peripheral tissues (17). However, the role of M-MDSCs in regulating immune functions during AT inflammation and whether there is cross-talk between adipocytes and M-MDSCs during obesity remains unclear to date. Thus, understanding the causal relationship between M-MDSCs and obesity-associated disorder will be critical for the therapeutic targeting of AT inflammation and obesity.

The murine CD33-related sialic acid-binding immunoglobulin-like lectins (siglecs) subfamily member siglec-E is primarily expressed in cells of myeloid origin, including macrophages, neutrophils, and dendritic cells (DCs) (18). Sialylation is a hallmark in immune regulation and functions as a key immunosuppressive checkpoint in various diseases, including allergies, autoimmune diseases, and neurodegeneration (19). The TNF receptor-associated factor 3 (TRAF3), a cytoplasmic adaptor protein and an important component of toll-like receptor (TLR) signaling, inhibits chronic inflammation and pathogenic infection in macrophages (20). Myeloid cell-specific TRAF3-deficient mice (M-TRAF3^-/-^) exhibit spontaneous chronic inflammation. TLR4 induces siglec-E expression in a MyD88-specific manner and siglec-E interacts with TLR4 to negatively regulate downstream TLR4 signaling (21, 22). TRAF3 is a well-known negative regulator of NF-κB-mediated proinflammatory signaling (23). TRAF3 activates signaling through myeloid differentiation factor 88 (MyD88) and TIR domain-containing adaptor inducing interferon-β, leading to the activation of interferon regulatory factor 3 (IRF3) (24). During obesity, TRAF3 switches into a pro-inflammatory phenotype that exacerbates metabolic dysfunction and insulin resistance (25). T-helper (Th) 2, and regulatory T (Treg) cells promote the activation of macrophages into a proinflammatory phenotype (26). However, it remains unclear whether TRAF3 and TLR4 signaling is associated with siglec-E that alters AT microenvironment to mediate obesity and AT inflammation.

Therefore, in this study, we will test a hypothesis of whether modulating siglec-E pathways can protect mice from AT inflammation during obesity. We will explore the role of siglec-E in HFD-induced obesity and related AT inflammation using siglec-E KO and WT C57BL/6 mice. Here we present the data identifying siglec-E as an essential regulator of M-MDSCs and CXCR3 expressing CD8^+^ T cells attendant functions through TRAF3 and Akt signaling in AT. These results may have implications for the physiology and pathophysiology associated with AT inflammation and metabolic diseases.

## Materials and methods

### Mice

Male WT C57BL/6 mice (aged 7 weeks) were purchased from the Jackson Laboratories (Bar Harbor, ME), and siglec-E knockout mice (siglec-E KO) were bred and verified in our laboratories at UTHSC (38). This study examined only male animals, as obesity is reported to be more prevalent in male mice as compared to females. All animals were maintained in the specific pathogen-free animal facility at UTHSC and housed at 20-22°C on a 12-hour light/dark cycle. After a week of acclimatization, animals were fed either a normal diet (ND) containing 10% calories as fat (cat no. D12450J, Research Diets, New Brunswick, NJ, USA) or a high-fat diet (HFD) containing 60% calories as fat (cat no. D12492, Research Diets, New Brunswick, NJ, USA **Tables 1**, **2**). Each diet was continued based on the experimental plan of 12 weeks. The body weight of each mouse and food consumption were monitored every week.

**Table 1:** Experimental models: organisms/strains and diets.

Reagent or resource	Source	Identifier
C57BL/6 mice (WT)	The Jackson Laboratory	Strain no. 000664
C57BL/6 mice (Siglec-E knockout)	Dr. Jianxiong Jiang’s Lab	N/A
Normal diet (ND), with 10% calories as fat	Research Diets	Cat. no. D12450J
High-fat diet (HFD), with 60% calories as fat	Research Diets	Cat. no. D12492

N/A, Not applicable.

**Table 2:** Chemicals, peptides, and recombinant proteins.

Reagent or resource	Source	Identifier
Red blood cell (RBC) lysis buffer, 1x	ThermoFisher Scientific	Cat. no. 00-4333-57
RIPA buffer	Alfa Aesar	Cat. no. J63306
Fetal bovine serum (FBS)	Genesee Scientific	Cat. no. 25-550H
Collagenase	Sigma-Aldrich	Cat. no. C6885
Roswell Park Memorial Institute (RPMI)- 1640 medium	Corning	Cat. no. 10-041-CV
Dulbecco’s modified Eagle medium (DMEM)	Corning	Cat. no. 10-013-CV
Ammonium-chloride-potassium (ACK) Lysing Buffer	ThermoFisher Scientific	Cat. no. A1049201
Paraformaldehyde, 4%	Alfa Aeser	Cat. no. J61899
BCA protein assay kit	ThermoFisher Scientific	Cat. no. 23225
Laemmli sample buffer, 4x	Bio-Rad	Cat. no. 1610747
2-β-mercaptoethanol	Alfa Aesar	Cat. no. A15890
Tris-buffered saline (TBS) containing 0.2% Tween 20	ThermoFisher Scientific	Cat. no. 28360
Polyvinylidene fluoride (PVDF) membranes	Bio-Rad	Cat. no. 1620174
Intercept blocking buffer	LI-COR Biosciences	Cat. no. 927-60001
Qiazol	Qiagen	Cat. no. 79306
Lipopolysaccharide	Sigma	Cat. No. L4391
Recombinant Mouse M-CSF	BioLegend	Cat. No. 576406
Recombinant Mouse IFN-γ	BioLegend	Cat. No. 575306
Penicillin-Streptomycin	Gibco	Cat. No. 15140-122

### Tissue isolation

At the end of each experiment, ND- or HFD-fed mice were euthanized using isoflurane followed by cervical dislocation. Spleens, mesenteric lymph nodes (MLNs), and epididymal white adipose tissue (eWAT) were isolated as we described previously, harvested, weighed, frozen in liquid nitrogen, and stored at -80°C until further processing. All the tissues were used for either biochemical or histological analyses.

### Metabolic parameters assessment

Blood glucose levels without fasting were examined at the endpoints of each study using a glucometer (Accu-Check Performa, Roche Diagnostics, Mannheim, Germany). The detailed methods are described in our previous publication (9).

### Single-cell suspension from spleens and MLNs

Spleens and MLNs from WT and siglec-E KO mice fed on ND or HFD were dissociated using a stomacher (Seward Stomacher^®^ 80) to produce single-cell suspensions. Red blood cells (RBCs) were eliminated by using an appropriate amount of RBC lysis buffer (cat. no. 00–4333–57, Invitrogen, Waltham, MA). The cells were collected by centrifugation (300 x g for 8 min), the supernatant was discarded, and the cell pellets were suspended in Roswell Park Memorial Institute (RPMI)−1640 medium (cat. no. 10–041-CV, Corning, NY) containing 10% fetal bovine serum (FBS; cat. no. 25–550 H, Genesee Scientific, San Diego, CA). Cell debris was removed using a 70μ filter and the cells were further processed for the subsequent experiments.

### Isolation of stromal vascular fraction cells from the AT

The e-WAT of WT and siglec-E KO mice fed on ND and HFD were minced, then chopped and digested with collagenase (1.5 mg/mL, Sigma-Aldrich, St. Louis, MO, USA; cat. no. C6885) in Dulbecco’s Modified Eagle Medium (DMEM) supplemented with 10% FBS during a 30-40 min incubation at 37°C with occasional shaking. Afterward, the cells were passed through a 100 μm cell strainer, centrifuged (800 x g for 8 min), and the resulting pelleted stromal vascular fractions (SVF) cells were collected. These SVF cells were resuspended in ammonium chloride-potassium (ACK) lysis buffer (cat. no. A1049201), incubated on ice for 4-5 min, collected by centrifugation (800 x g for 8 min), and the cell pellets were resuspended in RPMI with 10% FBS and processed for further analysis.

### Isolation and culture of BMDMs

To generate BMDMs, bone marrow flushed from mouse femurs and tibias were plated for 7 days in RPMI media (Cat. no. 10-041-CV, Corning, NY) supplemented with 1% sodium pyruvate, 1% HEPES, 10% heat-inactivated FBS (Cat. No. 25–550 H, Genesee Scientific, San Diego, CA) 1% penicillin/streptomycin (Cat. No. 15140-122, Gibco, MT, USA) 0.05 mm β-mercaptoethanol (Cat. No. A15890, Alfa Aesar, MA, USA). To obtain differentiated macrophages, 5 ng/ml of macrophage colony stimulating factor (M-CSF) (Cat. No. 576406, BioLegend, San Diego, CA) was added every 2 d for 6 d. BMDMs were stimulated with 10 ng/ml LPS (Cat. no. L4391, Millipore Sigma, St. Louis, MO) and 50 ng/ml IFNγ (Cat. No. 575306, BioLegend, San Diego, CA) to promote M1 polarization. Cells were rested in RPMI containing 10% FBS and 1% pen-strep for 24 hours before using in experiments.

### AT histology and hematoxylin & eosin staining

At the experimental endpoint, the e-WAT from WT and siglec-E KO mice fed on ND and HFD were fixed in 4% paraformaldehyde and paraffin-embedded. Tissue sections (4 μm thickness) were deparaffinized in xylene, rehydrated by decreasing alcohol gradation, and stained with hematoxylin and eosin (H&E). The sections were briefly stored in 95% ETOH, placed in xylene for clearing, and coverslips were added. Images of H&E-stained tissues were captured under an Olympus BX43 bright field microscope (Japan) with camera DP28 using a 10X or 20X objective.

### Quantification of H&E-stained AT sections

The identification of adipocytes and nuclei from H&E-stained sections was performed using an artificial intelligence (AI)-based workflow that consisted of training on the AI model (Apeer, www.apeer.com, Zeiss) and using the resulting model to quantify image data offline (Arivis Vision4D, Zeiss). Approximately 6-20 objects that included adipocytes, nuclei, and background were annotated on 12 representative images to establish ground truth for training the AI model. For the quantification of the images, the AI model was used to annotate each image for adipocytes, nuclei, and background and to generate statistics on object size (μm^2^) and the number of objects per field. All images were filtered to remove incomplete or poorly annotated objects near the edge of the field of view.

### Flow cytometry analysis

At the experimental endpoint, the spleens, MLNs, and eWAT from WT and siglec-E KO mice fed on ND or HFD were collected and SVF cell suspensions were made as described above. Pools of ~1 x 10^6^ single cells were pelleted and resuspended in 80μL of ice-cold flow cytometry staining buffer (FACS buffer, PBS containing 1% FBS). BMDMs are either pretreated with M-CSF in the presence or absence of LPS and IFNγ and kept overnight at 37°C. For extracellular marker staining, cell suspensions as well as BMDMs were incubated with specific surface antibodies (30 min at 4°C). Fluorescent-conjugated antibodies and compensation beads were obtained from Bio-Legend (San Diego, CA). The following antibodies were used for this study (**Table 3**): PE-conjugated anti-mouse/human CD11b Ab, clone M1/70, cat. no. 101208; Alexa Fluor^®^ 488-conjugated anti-mouse CD11c Ab, clone N418, cat. no. 117311; APC-conjugated anti-mouse CD3 Ab, clone 17A2, cat. no. 100236; APC-conjugated anti-mouse CD4 Ab, clone GK1.5, cat. no. 100412; FITC-conjugated anti-mouse CD8a Ab, clone 53-6.7, cat. no. 100706; PE-conjugated anti-mouse CD183 (CXCR3) Ab, clone CXCR3-173, cat. no. 126506; APC-conjugated anti-mouse Ly-6G/Ly-6C (Gr-1) Ab, RB6-8c5, cat. no. 108412; APC/Fire™ 750-conjugated anti-mouse Ly-6C Ab, clone HK1.4, cat. no. 128046; and PE-conjugated anti-mouse NK-1.1 Ab, clone PK136, cat. no. 108708; BV421-conjygated anti-mouse F4/80, clone BM8, Cat. no. 123137; PE-conjugated anti-mouse NOS2, clone W16030C, Cat. no. 696806.

**Table 3:** Antibodies for flow cytometry.

Reagent or Resource	Clone	Source	Identifier
PE anti-mouse/human CD11b Ab	M1/70	BioLegend	Cat. no. 101208
Alexa Fluor^®^ 488 anti-mouse CD11c Ab	N418	BioLegend	Cat. no. 117311
APC anti-mouse CD3 Ab	17A2	BioLegend	Cat. no. 100236
APC anti-mouse CD4 Ab	GK1.5	BioLegend	Cat. no. 100412
FITC anti-mouse CD8a Ab	53-6.7	BioLegend	Cat. no. 100706
PE anti-mouse CD183 (CXCR3) Ab	CXCR3-173	BioLegend	Cat. no. 126506
APC anti-mouse Ly-6G/Ly-6C (Gr-1) Ab	RB6-8c5	BioLegend	Cat. no. 108412
APC/Fire™ 750 anti-mouse Ly-6C Ab	HK1.4	BioLegend	Cat. no. 128046
PE anti-mouse NK1.1 Ab	PK136	BioLegend	Cat. no. 108708
Brilliant Violet 421 anti-mouse F4/80	BM8	BioLegend	Cat. no. 123137
PE anti-mouse NOS2	W16030C	BioLegend	Cat. no. 696806

### Fluorescence-activated cell sorting

Isolated AT SVF were stained with anti-CD11b (BioLegend, San Diego, CA) and anti-Gr-1 (BioLegend, San Diego, CA) antibodies for MDSCs, and using flow cytometry. After identifying the MDSCs phenotype, M-MDSCs (CD11b^+^Gr-1^+^Ly6c^hi^Ly6g^lo^) were sorted (98% pure) using the FACSAriaTM cell-sorter system (BD-PharMingen, CA).

### Multiplex assays

After euthanization, mice were subjected to cardiac puncture for collection of blood, which was then allowed to clot for 30 min at room temperature before centrifugation for 10 min and collection of serum. The serum was stored at −80°C until further analysis. Measurement of inflammation-associated biomarkers was performed using a Milliplex MAP Mouse Cytokine/Chemokine Magnetic Bead Panel Premixed 25 Plex Immunology Multiplex Assay kit (Cat. no. MCYTOMAG-70K-PMX, Millipore Sigma). We analyzed the serum samples for the following targets: granulocyte macrophage colony-stimulating factor (GM-CSF), MCP1, IL-12, IL-5, IL-2, IL-13, IL-1β and IL-17. Briefly, each well of the assay plate was wetted with buffer and 25 μl beads were added to each. In blank wells, we added 25 μl of assay buffer, while in sample wells serum sample was added, and the plate was shaken in the dark at 4°C overnight. The next day, each well was washed twice with buffer, a 25 μl detection antibody was added to each well, and the plate was shaken in the dark at RT for 1 hr. Then 25 µl streptavidin-phycoerythrin (PE) was added to each well, the plate was shaken in the dark at RT for 15 min, and then the amplification buffer was added to the wells. After that plate was incubated as above for an additional 15 min and then streptavidin PE/amplification buffer was removed, beads were resuspended in 150 µl assay buffer and analyzed using a Luminex™ System (Austin, TX) and software from Bio-Rad (Hercules, CA). The results were expressed as mean fluorescence intensity (MFI).

### Immunoblotting

At the experimental endpoint, the eWAT from WT and siglec-E KO mice fed on ND or HFD was collected and quickly frozen in liquid nitrogen before further processing. Tissue lysates were prepared using RIPA buffer (Cat. no. J63306, Alfa Aesar, Haverhill, MA) with protease and phosphatase inhibitors. The resulting lysates were subjected to centrifugation (16000 x g for 20 min) to remove cell debris, the supernatant was collected and the protein concentration in each lysate was measured using a BCA protein assay kit (Cat. no. 23225, Thermo Fisher Scientific, Waltham, MA). An equal amount of protein in each lysate was mixed with 4X Laemmli sample buffer (Cat. no. 1610747, Bio-Rad, Hercules, CA) containing fresh 2-β-mercaptoethanol (Cat. no. A15890, Alfa Aesar, Haverhill, MA), and heated at 95°C for 5 min. The protein samples were subsequently separated on a 10% sodium dodecyl sulfate (SDS)-polyacrylamide gel (Bio-Rad, Hercules, CA). After electrophoresis, proteins were transferred to polyvinylidene fluoride (PVDF) membranes (Cat. no. 1620174, Bio-Rad, Hercules, CA) by using a Trans-Blot Turbo instrument (Bio-Rad, Hercules, CA). Membranes were blocked with intercept blocking buffer (cat. no. 927-60001, LI-COR Biosciences, Lincoln, NE) at room temperature for 1-2h and incubated with antibodies specific for TRAF3 (monoclonal Ab, cat. no. 66310-1-Ig, Proteintech), Akt2 (clone 8B7, cat. no. SC-81436, Santa Cruz Biotechnology), or Akt1/2/3 (clone BDI111, cat. no. SC-56878, Santa Cruz Biotechnology) overnight at 4°C with continuous shaking (**Table 4**). Later, the membranes were washed three times (5-10 min. each) with TBS containing 0.2% Tween 20 (cat. no. 28360, Thermo Fisher Scientific, Waltham, MA). The membranes were then incubated at RT for 1 hr with the appropriate secondary antibodies (1:5,000), namely IRDye^®^ 800C W-labeled goat anti-mouse IgG (cat. no. 926-32210, LI-COR Biosciences, Lincoln, NE), IRDye^®^ 680RD-labeled goat anti-mouse IgG (cat. no. 925-68070, LI-COR Biosciences), or goat anti-rabbit IgG (cat. no. 926–32211, LI-COR Biosciences), then washed three times (5-10 min. each) with TBS containing 0.2% Tween 20 (cat. no. 28360, Thermo Scientific Fisher, Waltham, MA). Images were taken with an LI-COR Odyssey^®^ DLX imaging system (LI-COR Biosciences, Lincoln, NE) and densitometric analyses were performed using ImageJ software (NIH).

**Table 4:** Antibodies for immunoblotting.

Reagent or resource	Source	Identifier
TRAF3 monoclonal Ab	Proteintech	Cat. no. 66310-1-Ig
NF-kB (P65) (F-6)	Santa Cruz Biotechnology	Cat. no. SC-8008
MyD88 (E-11)	Santa Cruz Biotechnology	Cat. no. SC-74532
TLR4 (25)	Santa Cruz Biotechnology	Cat. no. SC- 293072
β-Actin rabbit monoclonal Ab	LI-COR Biosciences	Cat. no. 926-42210
β-Actin mouse monoclonal Ab	LI-COR Biosciences	Cat. no. 926-42212
Akt2 Ab (8B7)	Santa Cruz Biotechnology	Cat. no. SC-81436
Akt1/2/3 Ab (BDI111)	Santa Cruz Biotechnology	Cat. no. SC-56878
IRDye^®^ 800CW-conj goat anti-mouse IgG	LI-COR Biosciences	Cat. no. 926-32210
IRDye^®^ 680RD-conj goat anti-mouse IgG	LI-COR Biosciences	Cat. no. 925-68070
Goat anti-rabbit IgG	LI-COR Biosciences	Cat. no. 926–32211

### RNA isolation and RT-qPCR analysis

Total RNA was isolated from the AT of WT and siglec-E KO mice fed on ND or HFD and using Qiazol (QIAGEN, Germantown, MD) following the manufacturer protocol. In addition, total RNA from the purified isolated M-MDSC was isolated by RNeasy Mini Kit (QIAGEN, Germantown, MD) following the manufacturer protocol. The concentration and purity of the total RNA were determined using a Nanodrop spectrophotometer (Thermo Fisher Scientific, Waltham, MA). Total RNA (1μg) was transcribed into first strand cDNA using an iScript cDNA synthesis kit (Bio-Rad, Hercules, CA) according to the manufacturer’s procedure. Expression of siglec-E and GAPDH mRNAs were measured by quantitative PCR (qPCR) using the appropriate primers with an iTaq Universal SYBR Green Supermix (Bio-Rad, Hercules, CA) on a CFX96 Touch™ Real-Time PCR detection system (Bio-Rad, Hercules, CA). GAPDH (QIAGEN, Germantown, MD) was used as a reference gene. The primers used in the experiment are listed in **Table 5**.

**Table 5:** List of primer sequences used for RT-qPCR.

Gene	Sequence (5’ to 3’)	Source
Siglec-E	F: GTGTCCACAAGAATGACCATCCG	IDT
	R: TGAGCCATTCTTCAGGATTGTGG	
Arg1	F: CATTGGCTTGCGAGACGTAGAC	
	R: GCTGAAGGTCTCTTCCATCACC	
KLF4	F: CTATGCAGGCTGTGGCAAAACC	
	R: TTGCGGTAGTGCCTGGTCAGTT	
IFN-γ	F: GACCTAGAGAAGACACATCAG	
	R: AACAGCCATGAGGAAGAG	
CD11c	F: TGCCAGGATGACCTTAGTGTCG	
	R: CAGAGTGACTGTGGTTCCGTAG	
NF-κB	F: GAAGACACGAGGCTACAA	
	R: GGAAGGCATTGTTCAGTATC	
STAT3	F: AGGAGTCTAACAACGGCAGCCT	
	R: GTGGTACACCTCAGTCTCGAAG	
C/EBPα	F: CAGGAGGAAGATACAGGAAG	
	R: AGGACACAGACTCAAATCC	
FASN	F: CACAGTGCTCAAAGGACATGCC	
	R: CACCAGGTGTAGTGCCTTCCTC	
PPARγ	F: CCAAAGTGCGATCAAAGTAG	
	R: CCATGAGGGAGTTAGAAGG	
Resistin	F: GGGAATTGTGTGGGAAATG	
	R: GAGAGTCTCAAAGAGGAAGG	
GAPDH	F: GAAGCCCATCACCATCTT	
	R: CAGTAGACTCCACGACATAC	

Primers were purchased from IDT, Coralville, IA, USA.

Arg1, Arginase 1; KLF4, Kruppel-like factor 4; IFN-γ, Interferon-gamma; Gapdh, Glyceraldehyde-3-phosphate dehydrogenase; NF-kB, nuclear factor kappa-light-chain-enhancer of B cells; Stat, Signal transducer and activator of transcription; FASN, fatty acid synthase; PPARγ; Peroxisome proliferator-activated receptor gamma.

### RNA-seq analysis

eWAT was collected from the ND and HFD-fed mice and SVF cells were isolated as described earlier. Immune cells were isolated from SVF of both ND and HFD-fed mice using CD45 Microbeads (Cat. No. 130-052-301, Miltenyi Biotec, Bergisch Gladbach, Germany). Qiazol (QIAGEN, Germantown, MD) extraction of total RNA was performed according to the manufacturer’s instructions. The concentration and purity of the RNA were determined using a Nanodrop spectrophotometer (Thermo Fisher Scientific, Waltham, MA). Samples were shipped to Cincinnati Children’s Hospital core facility for further RNA-seq analysis. RNA libraries for RNA-seq were prepared using Illumina stranded mRNA Library Prep Kit following the manufacturer’s protocols. Sequence reads were trimmed using Trim Galore! v0.4.2 and cutadapt v1.9.1. The trimmed reads were aligned to the reference mouse genome version mm10 using STAR v2.6.1e. Aligned reads were stripped of duplicate reads with the program Sambamba v0.6.8. Read counting was done with the program featureCounts v1.6.2 from the Rsubread package. Raw counts were normalized as transcripts per million (TPM). The Raw data is submitted to Gene Expression Omnibus (GEO) accession number (GSE244406).

### Statistical analysis

All statistical analysis was performed using GraphPad Prism software (version 9.0; GraphPad Software Inc. **Table 6**). Data are expressed as the mean values ± standard error of the mean (SEM) for all experiments. Statistical significance was calculated in comparison to control groups and determined by one-way ANOVA or Student’s T-test depending on the experimental groups. A p-value of 0.05 was considered the level of significance in all analyses (ns [not significant] p > 0.05, * p < 0.05, ** p < 0.01, *** p < 0.001). Graphical representations were generated using GraphPad Prism software (GraphPad Software, Boston, MA).

**Table 6:** Software and websites.

Reagent or resource	Source	Identifier
Apeer software (www.apeer.com)	Zeiss	N/A
Arivis Pro (Vision4D) software	Zeiss	N/A
LI-COR Odyssey^®^ DLX imaging system	LI-COR Biosciences	N/A
ImageJ software	NIH	N/A
GraphPad Prism software (version 9.0)	GraphPad Software Inc.	N/A
Adobe Illustrator software (version 27.5)	Adobe Inc.	N/A

N/A, Not applicable.

## Results

### Siglec-E alters during HFD-induced AT inflammation

**Figure 1:**
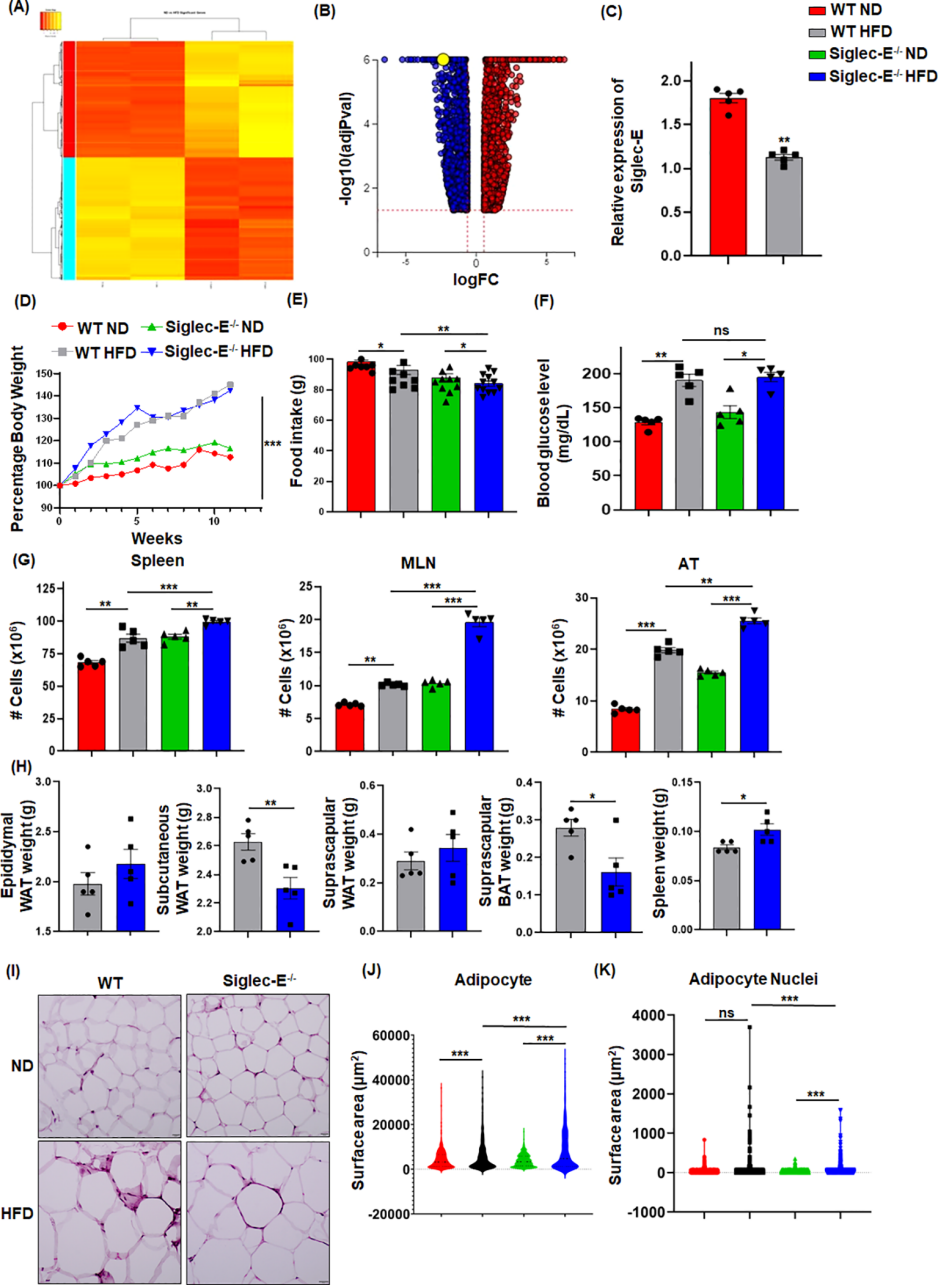
HFD-induced obesity downregulates Siglec-E in AT. **(A)** Heatmap of differentially expressed genes (DEGs) in ND and HFD-fed mice after 12 weeks of feeding. **(B)** Volcano plots of all DEGs, with siglec-E expression highlighted in yellow circle. **(C)** Relative expression of siglec-E in ND and HFD-fed WT animals after 12 weeks of feeding. **(D)** Comparison of percentages of body weight changes between WT and siglec-E KO mice during ND and HFD induction. **(E)** Food consumption of ND and HFD-fed WT and siglec-E KO mice after 12 weeks of feeding. **(F)** Blood glucose levels in ND and HFD-fed WT and siglec-E KO mice. **(G)** A total number of cell populations in the spleen, mesenteric lymph nodes (MLNs), and AT of ND and HFD-fed WT and siglec-E KO mice. **(H)** Total weight of epididymal white adipose tissue (eWAT), subcutaneous WAT, suprascapular WAT, suprascapular brown AT (BAT), and spleen of 12-week HFD-fed WT and siglec-E KO mice. **(I)** Representative pictures for H&E-stained epididymal AT sections collected from ND and HFD-fed WT and siglec-E KO mice. **(J, K)** Quantitative analyses of the H&E images. Data are expressed as mean ± SEM; from three independent experiments (n=5 mice per group). Statistical significance was calculated using either one-way ANOVA or Student’s t-test (ns, not significant; * p<0.05, ** p<0.01, *** p<0.001).

We performed RNA-seq analysis using CD45^+^ cells isolated from the eWAT of mice fed HFD or normal fat diet (ND) for 12 weeks to determine the alterations in gene expression. We noticed more than 1,000 genes whose expression was differentially regulated by HFD-induced obesity (**Figures 1A**; **Supplementary Figures S1A, S1B**). One of the major genes downregulated in mice fed HFD as compared to ND was siglec-E (**Figure 1B**). We validated this result using RT-qPCR analysis (**Figure 1C**). We designed our study to determine the effect of a decline in siglec-E expression in AT during HFD using WT and siglec-E KO mice (**Supplementary Figure S1C**). HFD increased the body weight of both WT and siglec-E KO mice compared to those fed ND (**Figures 1D**; **Supplementary Figure S1D**). Although WT HFD and siglec-E KO mice gain almost similar body weight, the food intake of the siglec-E KO mice was significantly lower than the WT mice (**Figures 1D, E**). The blood glucose levels of the HFD-fed siglec-E KO and WT mice were increased as compared to ND-fed WT and siglec-E KO mice (**Figure 1F**).

Next, we observed that HFD-fed siglec-E KO mice exhibited significantly increased cellularity in the spleen, mesenteric lymph nodes (MLNs), and AT as compared to similarly fed WT counterparts (**Figure 1G**). In addition, the deletion of siglec-E slightly increased the eWAT and suprascapular WAT weight (**Figure 1H**), while suprascapular brown AT and spleen weight is significantly decreased in siglec-E KO mice fed with an HFD (**Figure 1H**). AT histology with hematoxylin and eosin (H&E) staining shows that HFD-fed WT and siglec-E KO mice exhibited enhanced obesity-related characteristics, including the enlargement of adipocyte surface area and larger lipid droplets relative to the corresponding ND-fed mice (**Figure 1I**). When the surface area of the adipocytes and their nuclei were quantified using an artificial intelligence (AI)-based workflow, we observed a significant increase in the adipocyte surface area from the HFD-fed mice, relative to that in the ND-fed mice (**Figures 1J, K**; **Supplementary Figures S1E, S1F**). Interestingly, nuclei staining was comparatively much higher in the AT of HFD-fed siglec-E KO mice than in the HFD-fed WT mice (**Figures 1J, K**; **Supplementary Figures S1E, S1F**). Taken together, all these data suggest that the levels of metabolic markers expressed in siglec-E KO mice were more likely to be influenced by HFD-induced AT inflammation as compared to WT mice.

### Siglec-E deletion alters AT macrophages and dendritic cells during obesity

**Figure 2:**
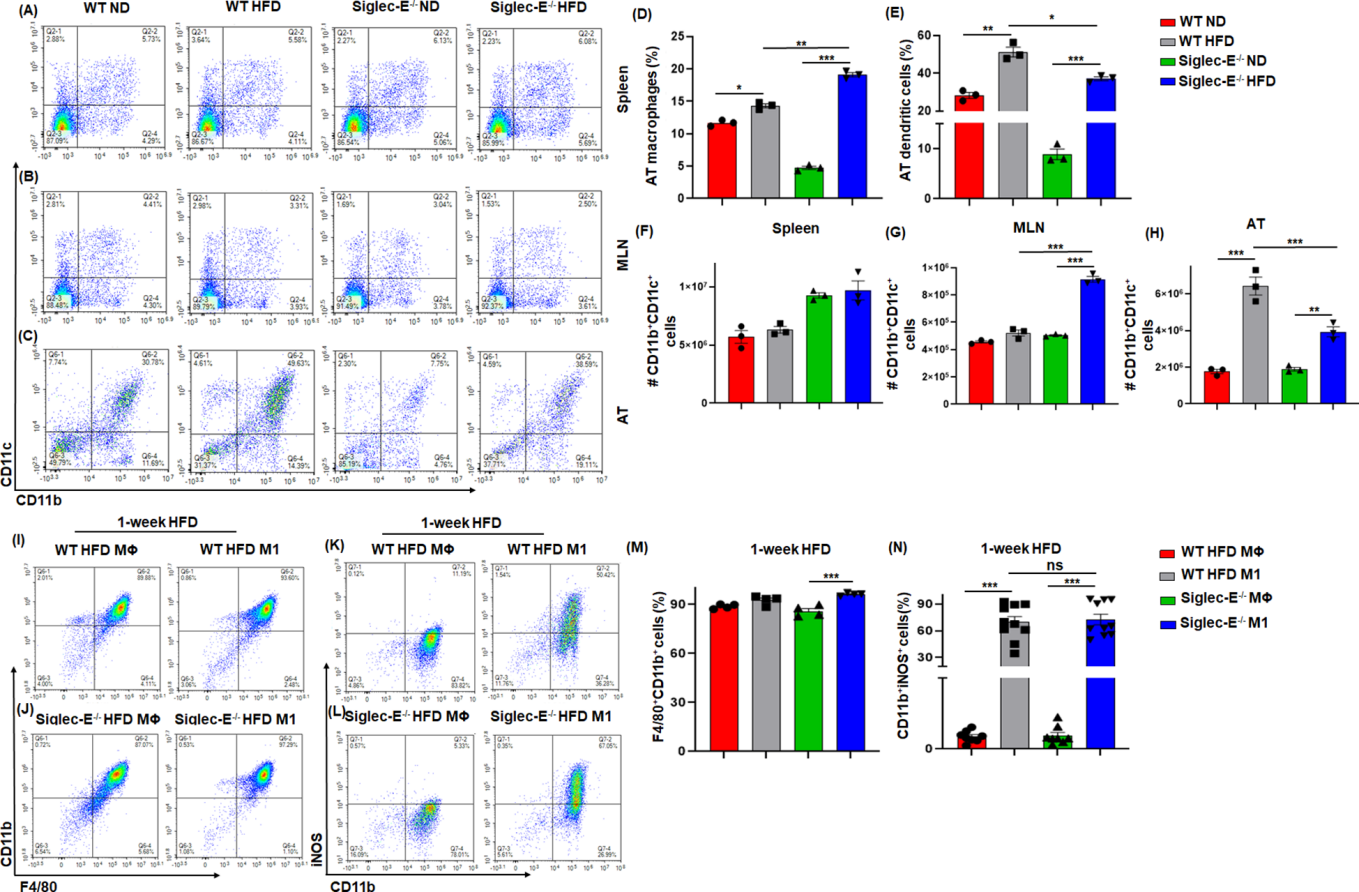
Siglec-E deletion increases the frequency of macrophages and dendritic cells (DCs) in AT. Representative flow cytometry plots of the macrophages (CD11b^+^, lower right quadrant) and DCs (CD11b^+^CD11c^+^, upper right quadrant) in the **(A)** spleen, **(B)** MLNs, and **(C)** AT of the ND and HFD-fed WT and siglec-E KO animals. The changes of the percentages of the macrophages **(D)**, and DCs **(E)** in ND and HFD-fed WT and siglec-E KO animals. **(F)** The total number of DCs in Spleen, **(G)** MLNs, and **(H)** AT in ND and HFD-fed WT and siglec-E KO animals. Representative flow cytometry plots of the **(I, J)** F4/80^+^CD11b^+^ and **(K, L)** CD11b^+^iNOS^+^ cell population from bone marrow-derived macrophages (BMDMs) polarized *ex vivo* to M1 macrophage phenotypes after 1-week HFD-fed WT and siglec-E KO mice. The changes of the percentages of the **(M)** F4/80^+^CD11b^+^ and **(N)** CD11b^+^iNOS^+^ cell population in MΦ and M1 macrophage after 1-week HFD-fed WT and siglec-E KO mice. Data are expressed as mean ± SEM; from three independent experiments (n=5 mice per group). Statistical significance was calculated using one-way ANOVA (ns, not significant; * p<0.05, ** p<0.01, *** p<0.001).

It has been shown that during obese conditions, AT is infiltrated by pro-inflammatory macrophages (M1) that maintain AT inflammation during obesity (27, 28). DCs are also crucial to bridge innate and adaptive immunity and facilitate the recruitment of macrophages during obesity (29). We investigated whether the absence of siglec-E altered the percentage and numbers of macrophages and DCs during HFD diet-induced AT inflammation. The macrophages in the spleen and MLNs of WT and siglec-E KO mice were not altered by HFD as compared to ND (**Figures 2A, B** lower right quadrants, **Supplementary Figures S2A, S2B**). However, the AT macrophage was higher in HFD compared to ND in both groups (**Figure 2C**, lower right quadrants and **Figure 2D**). Interestingly, the difference in the number of macrophages was significantly higher in siglec-E KO mice fed HFD as compared to ND (19.1% vs 4.76%) (**Figure 2D**). Next, we examined the percentage of DCs in the spleens, MLNs, and AT of both WT and siglec-E KO mice after HFD feeding. The percentage of DCs in the spleen and MLNs of HFD-fed WT and siglec-E KO mice were not altered compared to ND-fed mice (**Figure 2A**, upper right quadrants, **Supplementary Figures S2C, S2D**). However, HFD-fed WT and siglec-E KO mice exhibited a significant increase in DCs frequency in AT (**Figure 2C**, upper right quadrants, and **Figure 2E**). However, a slight decrease was observed in the number of DCs in spleens and MLNs (**Figures 2F, G**). In contrast, the number of DCs in AT was significantly decreased in HFD-fed siglec-E KO mice relative to those in HFD-fed WT mice (**Figure 2H**). Taken together, these data suggest that the absence of siglec-E contributes to enhancing the infiltration of macrophages and alters DCs. However, more detailed studies are required for a conclusion on the reduction of DCs in AT to a greater extent in siglec-E KO mice fed on HFD as compared to WT mice.

### Siglec-E polarizes bone marrow-derived macrophages toward M1 phenotypes

Next, we used bone marrow-derived macrophages (BMDMs) from both WT and siglec-E KO mice after 1 week of HFD feeding to characterize the macrophage phenotypes. The BMDMs were either pretreated with M-CSF in RPMI media (control differentiated macrophage (MΦ) or with M-CSF along with LPS and IFNγ to differentiate into (M1) phenotype. We notice a very slight difference between MΦ and M1 macrophages in both WT and siglec-E KO mice (**Figures 2I, J, M**) after 1 week of HFD feeding. However, the percentages of iNOS expressing M1 were significantly increased compared to MΦ macrophages after 1 week of HFD feeding (**Figures 2K, L, N**). Further, the percentages of differentiated M1 were significantly increased in siglec-E KO mice as compared to WT counterparts after 1 week of HFD feeding (**Figure 2N**). These data suggest that deletion of siglec-E induces the M1 inflammatory macrophage after HFD feeding, thus supporting AT inflammation after HFD induction.

### Siglec-E deficiency induces M-MDSCs in AT during obesity

**Figure 3:**
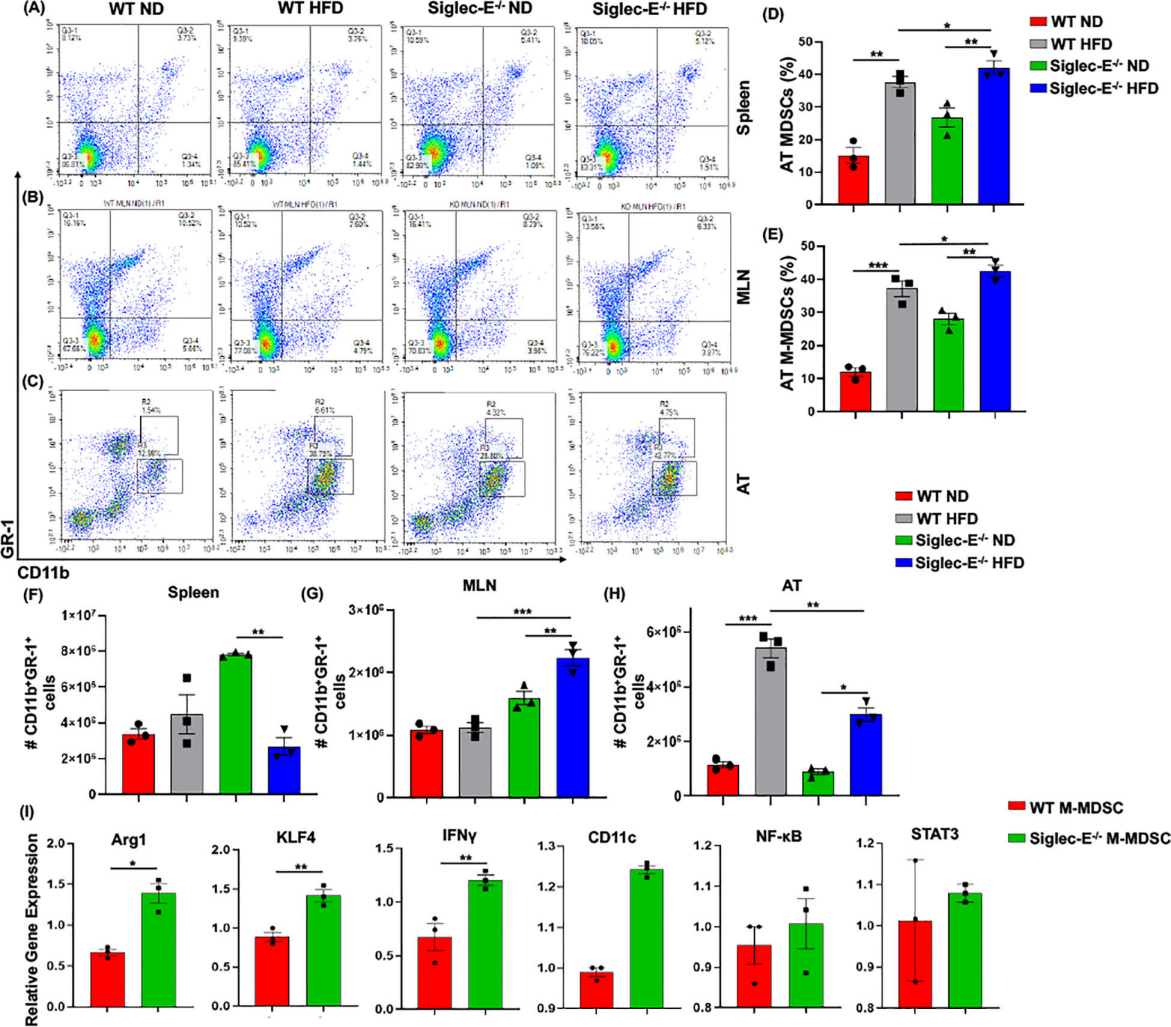
Siglec-E deletion induces M-MDSCs in AT during obesity. Representative flow cytometry plots for the MDSCs (CD11b^+^Gr-1^+^, upper right quadrant) in the **(A)** spleen, **(B)** MLNs of the ND and HFD-fed WT and siglec-E KO animals. Representative flow cytometry plots for the M-MDSCs (CD11b^+^-Gr-1^+^-Ly6G^lo^-Ly6c^hi^) and G-MDSCs (CD11b^+^-Gr-1^+^-Ly6G^hi^-Ly6c^lo^) in the AT **(C)** of the ND and HFD-fed WT and siglec-E KO mice. **(D, E)** The changes in the percentages of MDSCs and M-MDSCs in ND and HFD-fed WT and siglec-E KO mice. **(F)** The total number of MDSCs in Spleen, **(G)** MLN, and **(H)** AT in ND and HFD-fed WT and siglec-E KO animals. **(I)** Relative gene expression of Arg1, KLF4, IFNγ, CD11c, NF-κB, and STAT3 from AT-isolated purified M-MDSCs (98% pure) from the HFD-fed WT and siglec-E KO mice. Data are expressed as mean ± SEM; from three independent experiments (n = 5 mice per group). Statistical significance was calculated using either one-way ANOVA or Student’s T-test (* p<0.05, ** p<0.01, *** p<0.001).

The two forms of CD11b^+^Gr-1^+^ MDSCs (monocytic M-MDSCs and granulocytic G-MDSCs) have been functionally characterized in the peripheral tissue (17), the role of MDSCs in AT inflammation has not yet been studied in detail. In this study, we observed that in both WT and siglec-E KO mice, the accumulation of MDSCs in the spleen was slightly increased in the HFD-fed mice (**Figure 3A**, upper right quadrants, and **Supplementary Figure S3A**). In the MLNs, the percentage of MDSCs was significantly decreased in HFD-fed mice as compared with ND-fed mice, but this decrease was not statistically significant when comparing HFD-fed WT and siglec-E KO mice (**Figure 3B**, upper right quadrants and **Supplementary Figure S3B**). However, the percentage of MDSCs was significantly increased in the AT of HFD-fed WT and siglec-E KO mice relative to their ND-fed counterparts (**Figure 3D**). Interestingly, HFD-fed siglec-E KO mice exhibited greater numbers of MDSCs than HFD-fed WT mice (**Figure 3D**).

Next, we investigated the percentage and numbers of M-MDSCs (CD11b^+^Gr-1^+^Ly6G^lo^Ly6c^hi^) and G-MDSCs (CD11b^+^Gr-1^+^Ly6G^hi^Ly6c^lo^) in the spleen, MLNs, and AT. The percentage of M-MDSCs was significantly higher in the siglec-E KO mice fed with HFD relative to that in similarly fed WT mice (**Figure 3C**, upper gated quadrants, and **Figure 3E**). When we examined the relative numbers of MSDCs in the spleen, we did not observe any notable change in WT mice fed HFD versus ND (**Figure 3F**). In MLNs, the number of MDSCs was significantly changed after HFD feeding, but for both WT and siglec-E KO mice was comparatively lower in HFD-fed mice than in ND-fed mice (**Figure 3G**). In WT mice, the infiltration of MDSCs into the AT was significantly higher in HFD-fed mice than in ND-fed animals, while in siglec-E KO mice, we observed only a slight increase in MDSC numbers in the AT in HFD-fed mice than in ND-fed KO mice (**Figure 3H**). Interestingly, despite the reduced infiltration of MDSCs in HFD-fed siglec-E KO mice relative to that in HFD-fed WT mice (**Figure 3H**), we observed an increased percentage of MDSCs in the HFD-fed siglec-E KO mice than in their WT counterparts (**Figure 3D**). These results suggest that in the absence of siglec-E, the inflammatory responses are exacerbated by an increased percentage of M-MDSCs in the AT during HFD-induced AT inflammation.

Next, we determine the functions of M-MDSCs in siglec-E KO mice after HFD-induced obesity. We isolated and purified (98% pure by FACS AriaII cell sorter) M-MDSCs (CD11b^+^Gr-1^+^Ly6c^hi^Ly6g^lo^) from the stromal vascular fraction (SVF) of AT from HFD-fed WT and siglec-E KO mice. The RT-PCR analysis revealed that HFD-induction significantly increased the expression of Arg1, KLF4, and IFNγ expression in the M-MDSCs isolated from the HFD-fed siglec-E KO mice compared to the HFD-fed WT mice (**Figure 3I**). Further, the deletion of siglec-E also induced the expression of NF-κB, CD11c, and STAT3 in isolated M-MDSCs after HFD feeding, but the changes were not significant (**Figure 3I**). Thus, taking together data at our disposal suggests that M-MDSCs are a crucial factor in AT inflammation in the siglec-E KO mice. However, a precise adoptive transfer of M-MDSCs or depletion experiments is required for a prudent conclusion on the specific role of AT inflammation in siglec-E mice.

### Siglec-E deletion induces CXCR3 expressing CD8 T cells in the AT

**Figure 4:**
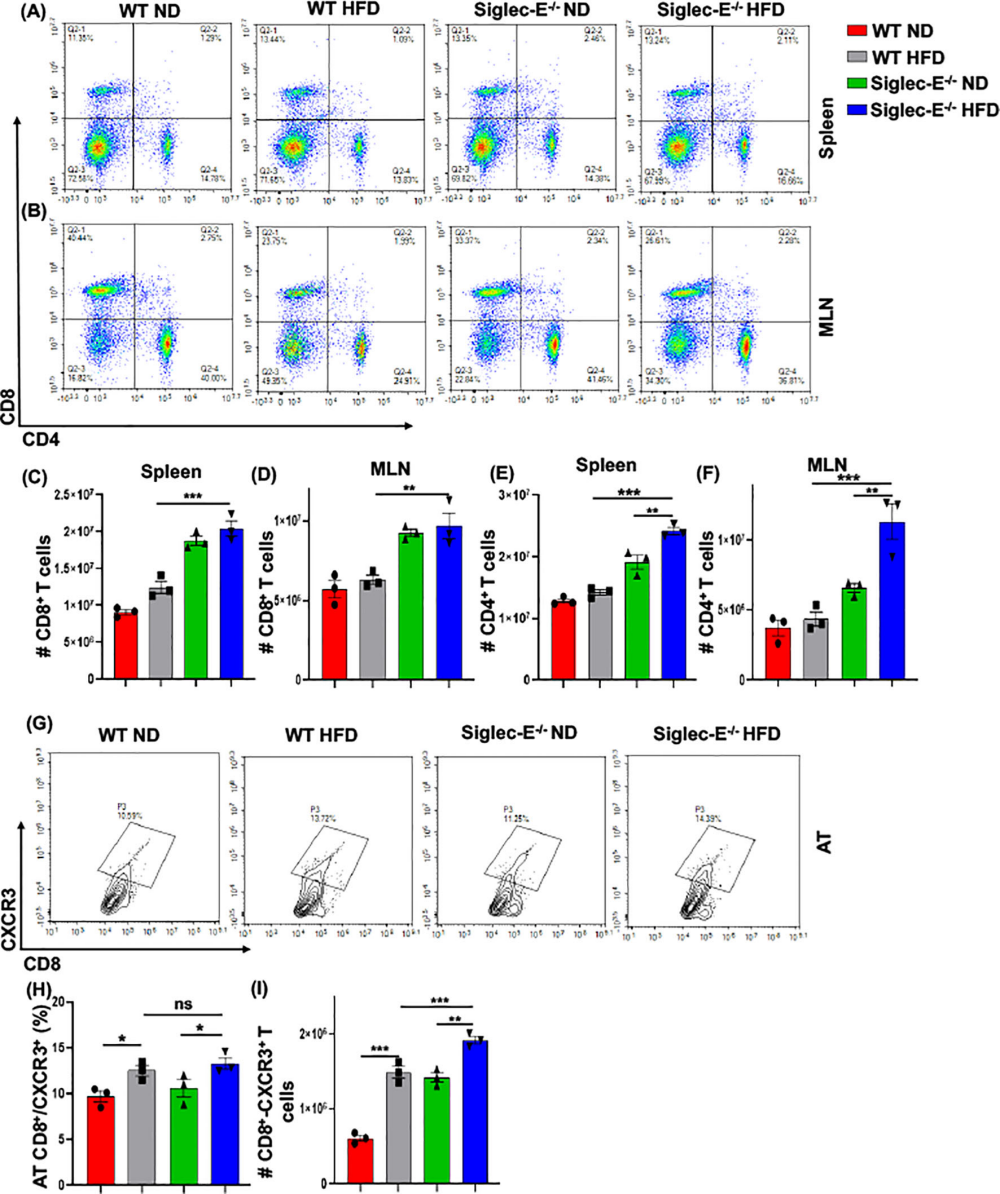
The absence of Siglec-E induced the frequency of AT CD8^+^CXCR3^+^ T cells. Representative flow cytometry plots of the CD4^+^ (lower right panel), CD8^+^ (top left panel), and CD4^+^CD8^+^ (top right panel) T cells in the **(A)** spleen, **(B)** MLNs of the ND and HFD-fed WT and siglec-E KO mice. The total number of CD8^+^ T cells in **(C)** spleen and **(D)** MLN of the ND and HFD-fed WT and siglec-E KO mice. The total number of CD4^+^ T cells in **(E)** spleen and **(F)** MLN of the ND and HFD-fed WT and siglec-E KO mice. **(G)** Representative flow cytometry images for the CD8^+^CXCR3^+^ T cells in ND and HFD-fed WT and siglec-E KO animals. **(H)** The changes of the percentages and **(I)** total number of CD8^+^CXCR3^+^ T cells in AT of the ND and HFD-fed WT and siglec-E KO mice. Data are expressed as mean ± SEM; from three independent experiments (n = 5 mice per group). Statistical significance was calculated using one-way ANOVA (ns, not significant; * p<0.05, ** p<0.01, *** p<0.001).

During obesity, macrophages propagate a pro-inflammatory microenvironment that recruits additional T cells, which eventually exacerbates AT inflammation during obesity (30). We observed an increased percentage of CD8^+^ T cells after HFD feeding in WT and siglec-E KO mice as compared to ND-fed mice, but the difference was not statistically significant (**Figure 4A**, upper left quadrants, and **Figure 4C**). We did not notice any major changes in the CD4^+^ T cells in either group of mice fed with ND or HFD (**Figure 4A**, lower right quadrants and **Figure 4E**). However, HFD-fed siglec-E KO mice exhibited a significantly increased percentage of CD4^+^ T cells in the spleen, relative to ND-fed KO mice (**Figure 4A**, lower right quadrants, **Figure 4E**) and significantly higher numbers of CD4^+^ T cells in the spleen relative to those detected in HFD-fed WT mice (**Figure 4E**). These results suggest that deletion of siglec-E is correlated in part with slightly increased T cell percentage in the spleen of HFD-induced obesity. In contrast, the percentage of CD8^+^ and CD4^+^ T cells was significantly decreased in MLNs in both WT and siglec-E KO mice fed HFD, relative to those of their ND-fed counterparts (**Figure 4B**, lower right quadrants, **Figures 4D, F**), the number of T cells was not much altered in mice of either group when fed HFD (**Figures 4D, F**). These results suggest that the altered T cells in the MLNs of HFD-fed siglec-E KO mice might alter obesity-associated inflammation.

CXCR3 expressing CD8^+^ T cells play potential roles in the function, and trafficking of T cells, induced after HFD feeding and for the development of effector T cells (9, 31, 32). Next, we examined whether the deletion of siglec-E alters the frequency of CD8^+^CXCR3^+^ T cells after HFD-fed WT and siglec-E KO mice. We observed an increased percentage (**Figures 4G, H**) and number (**Figure 4I**) of CD8^+^CXCR3^+^ T cells in the AT of HFD-fed WT and siglec-E KO mice relative to their ND-fed counterparts. Interestingly, the HFD-fed siglec-E KO mice exhibited increased percentages of CD8^+^CXCR3^+^ T cells in the AT compared to HFD-fed WT animals (**Figures 4G, H**). These results suggest that ablation of siglec-E is associated with higher recruitment of CXCR3-expressing CD8^+^ cells to the AT, which suggests the subsequent induction of AT inflammation in siglec-E KO mice.

### Deletion of siglec-E increases the percentage of neutrophils, NK, and NKT cells in AT

**Figure 5:**
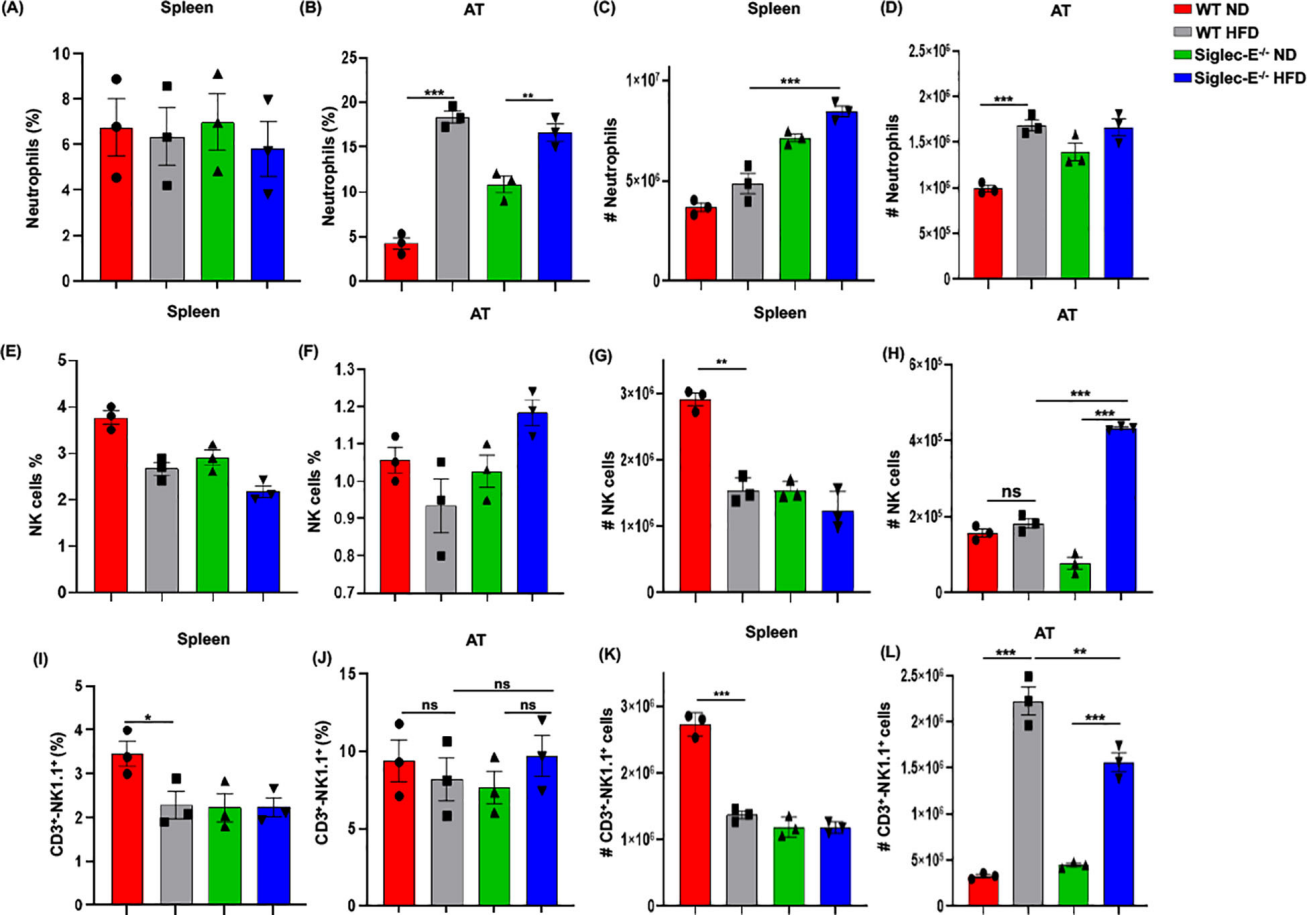
Siglec-E deletion elevates neutrophil, NK cell, and NKT cell counts in adipose tissue. The changes of the percentages of neutrophils in **(A)** spleen and **(B)** AT of ND and HFD-fed WT and siglec-E KO animals. The total number of neutrophils in **(C)** spleen and **(D)** AT of the ND and HFD-fed WT and siglec-E KO mice. The changes of the percentages of NK cells in **(E)** spleen and **(F)** AT of ND and HFD-fed WT and siglec-E KO animals. The total number of NK cells in **(G)** spleen and **(H)** AT of the ND and HFD-fed WT and siglec-E KO mice. The changes of the percentages of NKT cells in **(I)** spleen and **(J)** AT of ND and HFD-fed WT and siglec-E KO animals. The total number of NKT cells in **(K)** spleen and **(L)** AT of the ND and HFD-fed WT and siglec-E KO mice. Data are expressed as mean ± SEM; from three independent experiments (n = 5 mice per group). Statistical significance was calculated using one-way ANOVA (ns, not significant; * p<0.05, ** p<0.01, *** p<0.001).

HFD-induced obesity is associated with neutrophil chemotaxis that further attracts macrophages and other immune cells to the AT (33), and neutrophils are the first to infiltrate the site of inflammation (34). To determine whether neutrophils were altered in siglec-E KO after HFD feeding, we investigated their percentage in the AT of both WT and siglec-E KO mice. In the spleen, HFD induction did not alter much the percentage of neutrophils, although the cell numbers increased in both groups of HFD-fed mice (**Figures 5A, C**). The percentage of neutrophils in the AT was higher in HFD-fed WT mice than in their ND-fed littermates, but there were no significant differences in the percentage of neutrophils detected in siglec-E KO mice whether they were fed HFD or ND (**Figure 5B**). We also detected very slight changes in neutrophil frequency in HFD-fed siglec-E KO animals compared to their HFD-fed WT littermates (**Figure 5B**). Finally, the number of neutrophils infiltrating the AT in either group after HFD feeding was higher than that in their ND-fed littermates (**Figure 5D**). These data suggest that siglec-E deletion is also associated with increased AT infiltration of neutrophils, although further investigation is required for a prudent conclusion.

However, the number of NK cells significantly increased after HFD induction in the absence of siglec-E and it showed a significant increase compared to HFD-fed WT mice (**Figures 5E–H**). We observed a percentage of NKT cells in the spleen that was significantly lower in HFD-fed WT mice than in WT ND-fed mice, but there were no detectable noticeable changes in levels of splenic NKT cells in siglec-E KO animals fed either diet (**Figures 5I, K**). In contrast, the percentages of NKT cells in the AT HFD-fed siglec-E KO mice were significantly higher than in ND-fed siglec-E KO mice (**Figure 5J**). Furthermore, the number of NKT cells infiltrating the AT of HFD-fed siglec-E KO mice was significantly higher than in their ND-fed counterparts and comparatively lower than in HFD-fed WT mice (**Figure 5L**). These results suggest that the deletion of siglec-E also induces infiltration of NKT cells into the AT, which might contribute to the enhanced inflammatory processes observed during HFD-induced obesity.

### Siglec-E deletion induces TRAF3 and Akt expression in the AT

**Figure 6:**
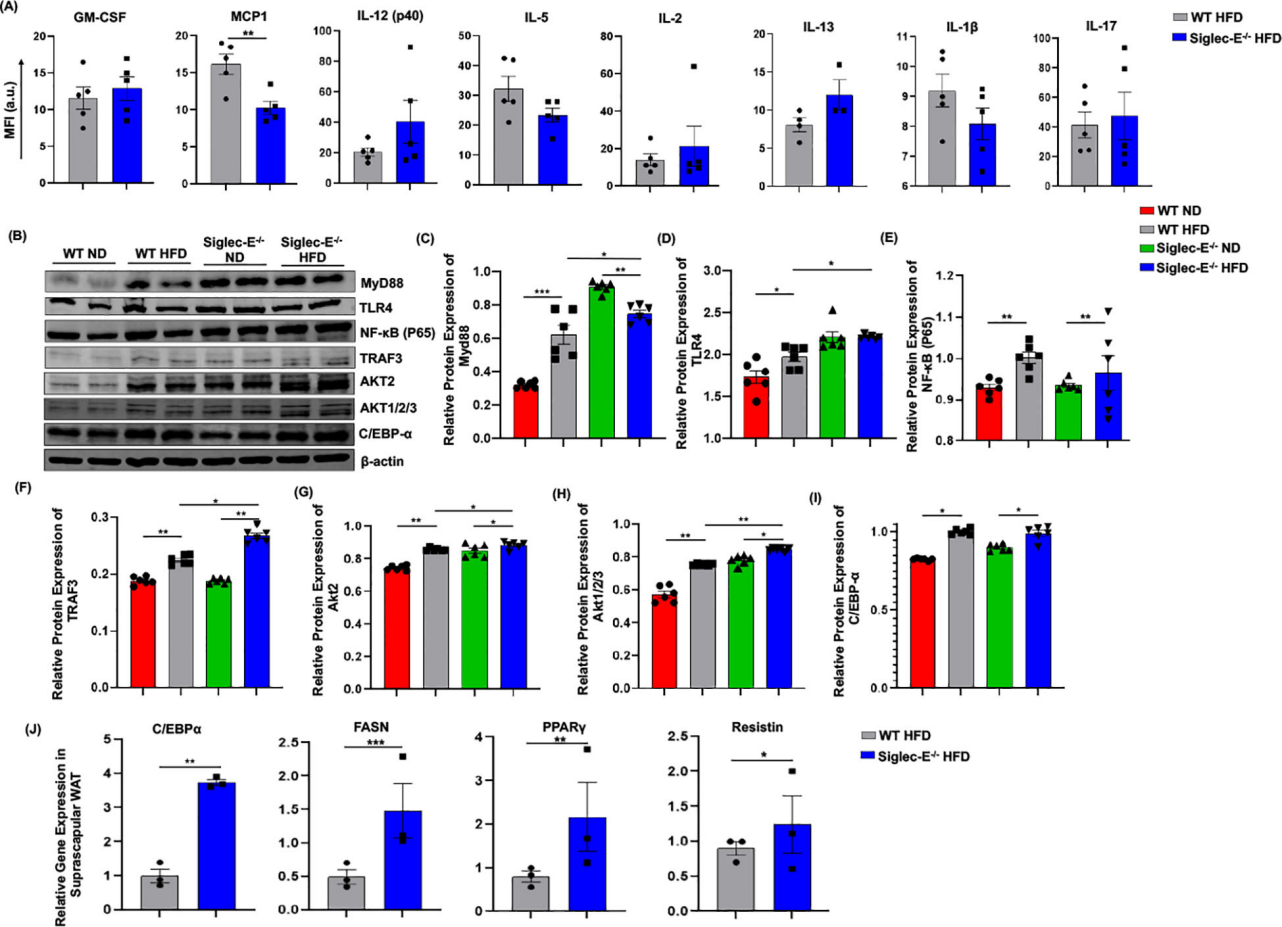
Deletion of siglec-E increased the expression of TRAF3 in the AT during HFD induction. **(A)** MFI of systemic cytokines, including GM-CSF, MCP1, IL-12 (p40), IL-5, IL-2, Il-13, IL-1β, IL-17 in HFD-fed WT and siglec-E KO mice. **(B)** Representative western blot images for MyD88, TLR4, NF-κB P65, TRAF3, AKT2, AKT1/2/3, C/EBPα, β-actin in the eWAT of ND and HFD-fed WT and siglec-E KO mice. Relative protein expression of **(C)** MyD88, **(D)** TLR4, **(E)** NF-κB P65, **(F)** TRAF3, **(G)** AKT2, **(H)** AKT1/2/3, **(I)** C/EBPα in the eWAT of ND and HFD-fed WT and siglec-E KO mice. **(J)** Relative expression of adipogenic genes, including C/EBPα, FASN, PPARγ, and resistin in the suprascapular WAT of 12-week HFD-fed WT and siglec-E KO mice. Data are expressed as mean ± SEM; from three independent experiments (n = 5 mice per group). Statistical significance was calculated using either one-way ANOVA or Student’s T-test (* p<0.05, ** p<0.01, *** p<0.001).

Adipocytes cross-talk with different immune cells induces systemic and local inflammation in AT during obesity. Thus, we compared the systemic inflammatory cytokines in the HFD-fed WT and siglec-E KO mice. The systemic level of MCP-1 is significantly decreased in HFD-fed siglec-E KO mice compared to the WT mice (**Figure 6A**). Although other cytokines including GM-CSF, IL-12 (p40), IL-2, and IL-17 showed an increasing trend in HFD-fed siglec-E KO mice compared to the WT mice, the increase was non-significant (**Figure 6A**). These results suggest that deletion of siglec-E might promote local inflammatory cytokines in obese AT.

It has been shown that siglec-E downregulates NF-κB expression in a Myd88-dependent manner. Next, we investigated the TLR4/NF-κB signaling during obesity and related inflammation in the absence of siglec-E. We noticed that in the absence of siglec-E Myd88 and TLR4 protein levels are increased in the AT compared to that of WT mice fed on both ND and HFD (**Figures 6B–D**). However, we did not observe any significant changes in the expression of NF-κB p65 in WT or siglec-E KO mice in response to HFD-feeding (**Figures 6B, E**), depicting that siglec-E-mediated AT- inflammation might not be associated with canonical NF-κB signaling pathway. We also investigated the adaptor protein of the non-canonical NF-κB signaling pathway. Since TRAF-3 functions as a non-canonical NF-κB activator during inflammatory signaling (35), we sought to determine whether and how deletion of siglec-E might affect TRAF-3 expression in mice under HFD conditions. We observed increased TRAF-3 expression in the AT of HFD-fed mice relative to their ND-fed counterparts (**Figures 6B, F**). Interestingly, HFD-fed siglec-E KO mice exhibited significantly higher levels of TRAF-3 than did HFD-fed WT mice (**Figure 6F**), suggesting that deletion of siglec-E contributes to inflammation through the TRAF3 signaling, which is a crucial adaptor protein of non-canonical NF-κB signaling.

Since Akt-regulated MDSC activities (16) and Akt2 signaling are required for M1 polarization of macrophages (36), we examined the effect of the siglec-E deletion on Akt protein expression. We detected increased expression of Akt1/2/3 and Akt2 in the AT in HFD-fed mice relative to the levels detected in ND-fed mice (**Figures 6G, H**). Importantly, the expression levels of Akt1/2/3 and Akt2 in the AT were significantly higher in siglec-E KO mice fed ND or HFD than those detected in the AT of HFD-fed WT mice (**Figures 6G, H**). These results suggest that ablation of siglec-E is associated with marked overexpression of Akt2, which might be a contributing factor during HFD-induced obesity. Further, we also investigated whether HFD feeding alters siglec-E KO mice adipogenic markers in the eWAT. Although HFD induction increased the expression of C/EBPα in the eWAT of both WT and siglec-E KO mice, HFD-fed siglec-E KO mice showed a non-significant decrease in the protein expression of C/EBPα compared to the HFD-fed WT mice (**Figure 6I**). To investigate the role of adipogenesis, we compared the adipogenic markers in the suprascapular WAT of HFD-fed WT and siglec-E KO mice. The results showed that deletion of siglec-E significantly increased the gene expression of adipogenic markers, including C/EBPα, PPARγ, FASN, and resistin in the suprascapular WAT of HFD-fed siglec-E KO mice compared to their WT counterparts (**Figure 6J**).

## Discussion

The factors that initiate AT inflammation during obesity remain elusive and are currently the subject of intense investigations. The present study is the first to demonstrate the role of siglec-E during HFD-induced AT inflammation by redistributing and increasing the number of M1 macrophage, CXCR3-expressing CD8^+^T cell, neutrophils, NK, and NKT cells while concomitantly reducing DCs in AT in genetically deficient siglec E mice. Furthermore, the absence of siglec-E prevalence in the inflammatory phenotype of M-MDSCs resulted in elevated expression of inflammatory markers that appeared to maintain low-grade chronic inflammation through the modulation of TRAF-3 and Akt signaling in AT. This compelling set of data provides new insight into siglec-E as a key contributing factor to controlling the AT inflammatory response and sustaining low-grade chronic inflammation in AT.

Siglec-E was first cloned in 2001 (37), and is a well-established lectin with diverse biological functions during bacterial infections, sepsis, cancer, and neurological disorders (38). Siglec-E is primarily expressed in the cells of the innate immune system (39), including macrophages and DCs, where it is involved in the regulation of TLR4-dependent cytokine production. Cross-linking siglec-E with specific antibodies decreases the expression of the inflammatory markers, namely TNF-α and IL-6, in BMDMs (40). Deletion of siglec-E serves as a negative regulator of neutrophil recruitment in the inflamed lung (41). In mice, siglec E enables DCs to induce Tregs leading to the inhibition of CD8^+^ T effector cells and reducing inflammatory conditions (42). After HFD feeding, we observed an increase in AT-resident CD8^+^CXCR3^+^ T cells in siglec-E KO mice, compared to WT mice, implicating that the deletion of siglec-E in AT inflammation leads to an exaggerated obesity-associated low-grade chronic inflammation. Altogether, this compilation of data demonstrates that siglec-E is functionally related to inflammatory signaling in AT inflammation during obesity.

AT inflammation is a crucial factor in the development of obesity and metabolic dysfunction (12). Macrophages and T cells are the most important cellular producers of pro-inflammatory cytokines. Excessive adipocyte hypertrophy leads to AT expansion, causes adipocyte death, and eventually recruits pro-inflammatory macrophages into the AT. In obesity, the proliferation of macrophages is highly abundant in AT, but not in other tissues, suggesting that the AT may provide a unique milieu for macrophages to facilitate obesity (43). Our data demonstrated an increased infiltration of macrophages in AT from both WT and siglec-E KO mice after feeding HFD, but the increase in macrophage infiltration was more pronounced in the siglec-E KO mice suggesting that siglec-E plays a role in the macrophage infiltration into the AT during diet-induced obesity. It is known that DCs recruit and activate macrophages at the site of inflammation and potentiate immune responses during HFD-induced obesity (44). They also integrate several triggering factors that promote homeostasis, immunity, inflammation, and an obesity-induced increase of CD11c^+^ DCs in the liver and AT (29, 45). In this study, we demonstrated that HFD induction increased the number of DCs in the AT under normal conditions in WT mice, which aligns with the findings of previous reports (46). However, we observed that siglec-E deficient mice exhibited a decreased frequency of DCs relative to WT mice under HFD conditions. Although genetic deletion of DCs can improve diet-induced obesity, there has been no demonstration of a lack of conventional CD11b^+^CD11c^+^ DCs related to obesity-related meta-inflammation (29, 47). Thus, the data at our disposal suggest that dysregulation in AT DCs in siglec-E-deficient, but not in WT mice signals the presence of an inflammatory condition through either reduction of Tregs and/or NKT cells function. However, identification of the key mechanisms by which DCs are dysregulated after the deletion of siglec-E during AT inflammation will require further investigation.

MDSCs are involved in the suppression of obesity-associated inflammation and their accumulation is associated with increased adiposity (17, 48) obesity was shown to be associated with increased accumulation of MDSCs in tumor-bearing mice (49). Accumulation of MDSCs in the AT and liver involves the expression of pro-inflammatory molecules including IL-6 and GM-CSF (17). In addition, the two MDSCs phenotypes M-MDSCs and G-MDSCs have distinct characteristics and functions. In HFD-induced obesity, the AT-derived expression of chemokine (C-C motif) ligand 5 (CCL5) enhances the local accumulation of pro-inflammatory M-MDSCs and subsequent inflammation via their cognate CCR5 receptors (50). During human obesity, monocytes shift toward a pro-inflammatory phenotype that might contribute to the development of low-grade inflammation and a population of immune-suppressive monocytes might contribute to the development of cancer in obesity (51). Under these conditions, an increase in M-MDSCs was observed in siglec-E KO mice versus WT mice, which could likely contribute to further progression toward AT inflammation. Furthermore, our cell sorting results suggested that the deletion of siglec-E significantly increased the inflammatory markers in M-MDSCs as compared to WT, which corroborated that siglec-E plays a crucial role in M-MDSCs inflammatory responses during obesity conditions. Thus, accumulation of M-MDSCs in siglec-E mice is associated with severe inflammation in AT.

The early immune cells to infiltrate the AT during obesity-induced inflammation are neutrophils, which secrete several inflammatory cytokines that shape adaptive immune responses (52, 53). The depletion of neutrophils is associated with improved insulin sensitivity (54). Our findings that the deletion of siglec-E, which acts as a negative regulator of neutrophil inflammation (41), led to increased infiltration of neutrophils in the AT. This subsequently led to the recruitment of other immune cells, such as NK and NKT cells, to promote AT inflammation. This corresponds with the previous notion that NK and NKT cells play differential roles in obesity and related metabolic diseases (33, 55, 56). It is clear from our findings that multiple immune cells are more susceptible to recruitment to the AT due to the HFD environment, particularly in the absence of siglec-E, to not only induce but exacerbate active inflammatory obesity-associated conditions.

The upregulation of siglec-E affects NF-κB responses in a MyD88-dependent manner, suggesting that its regulation of adipogenesis and obesity may occur via the NF-κB signaling pathway (40, 57). We noticed that the expression of TLR-mediated signaling protein is increased in the AT after deletion of siglec-E. Here, we also demonstrate that HFD feeding increased the expression of NF-κB in the AT. However, no significant difference in NF-κB expression was detected in siglec-E KO mice regardless of their fat diet, which suggests that the signaling pathways may be NF-κB independent. The known association of siglec-E with TLR4 signaling led us to investigate the effect of the siglec-E KO on different adapter molecules downstream of TLR4 signaling. Interestingly, we observed that deletion of siglec-E was associated with increased AT expression of TRAF3, which might recruit TRIF-associated signaling to control genes such as IRF3, and IRF7 that are associated with macrophage signaling (58). Our results suggest that the deletion of siglec-E might contribute to obesity-related inflammation by actively inducing the expression of TRAF3 in the AT. This is consistent with the observations that M-TRAF3_-/-_ mice exhibit spontaneous chronic inflammation and that deletion of TRAF3 in macrophages results in altered LPS-or polyinosinic-polycytidylic acid (polyI: C)-induced systemic responses (20). M-TRAF3_-/-_ mice experience spontaneous induction of inflammation, infection, and tumor formation at 15-22 months of age (20). In cultured macrophages, TRAF3 deletion increases the expression and secretion of pro-inflammatory molecules, including TNF-α, IL-1, IL-6, and IL-12 (58, 59). Thus, our data support the notion that the deletion of siglec-E might contribute to obesity-related inflammation. We also observed an extensive increase in the expression of Akt2 in siglec-E KO mice, relative to WT mice. Interestingly, mice with deletions of Akt2 and AMPKα2 exhibited effects that were protective against diet-induced obesity (60). Taken together, these findings suggest that siglec-E plays a crucial role in obesity-induced inflammation by controlling the expression of TRAF3 and Akt2 in the AT.

In summary, siglec-E has been identified as a central signaling hub to control pro-inflammatory immune cell activation, namely M-MDSCs, T cells, DCs, neutrophils, NK cells, and NKT in AT during HFD-fed conditions and limit their recruitment to AT and slow the onset towards diet-induced obesity. Our findings strongly demonstrate that siglec-E plays an adaptative role in protecting the adipocytes from inflammatory-mediated injury through reduced immune cell recruitment and TRAF3/Akt2 activity in AT during the early phase of obesity progression. This may open the door for new therapeutic development for siglec-E or its downstream signaling pathway to treat the deleterious effects of obesity.

## Data Availability

The original contributions presented in the study are included in the article/**Supplementary Material**. Further inquiries can be directed to the corresponding author.
