# Supplementary material for: Siglec-E augments adipose tissue inflammation by modulating TRAF3 signaling and monocytic myeloid-derived suppressor cells during obesity
Source: Front Immunol. 2025 Feb 4;16:1501307. doi: 10.3389/fimmu.2025.1501307 (PMC11832521; doi:10.3389/fimmu.2025.1501307)
Supplement: Supplementary file 1 [file DataSheet1.pdf]

Supplemental information

Siglec-E augments adipose tissue inflammation by modulating TRAF-3 signaling and monocytic myeloid-derived suppressor cells during obesity

Ahmed Rakib, Mousumi Mandal, Abdullah Al Mamun, Sonia Kiran, Nelufar Yasmen, Lexiao Li, Daniel Collier, Jianxiong Jiang, Frank Park, and Udai P Singh

Table S1. List of primers

Gene	Forward sequence	Reverse sequence
Siglec-E	GTGTCCACAAGAATGACCATCCG	TGAGCCATTCTTCAGGATTGTGG
C/EBPα	CAGGAGGAAGATACAGGAAG	AGGACACAGACTCAAATCC
PPARγ	CCAAAGTGCGATCAAAGTAG	CCATGAGGGAGTTAGAAGG
Resistin	GGGAATTGTGTGGGAAATG	GAGAGTCTCAAAGAGGAAGG
FASN	CACAGTGCTCAAAGGACATGCC	CACCAGGTGTAGTGCCTTCCTC
GAPDH	GAAGCCCATCACCATCTT	CAGTAGACTCCACGACATAC

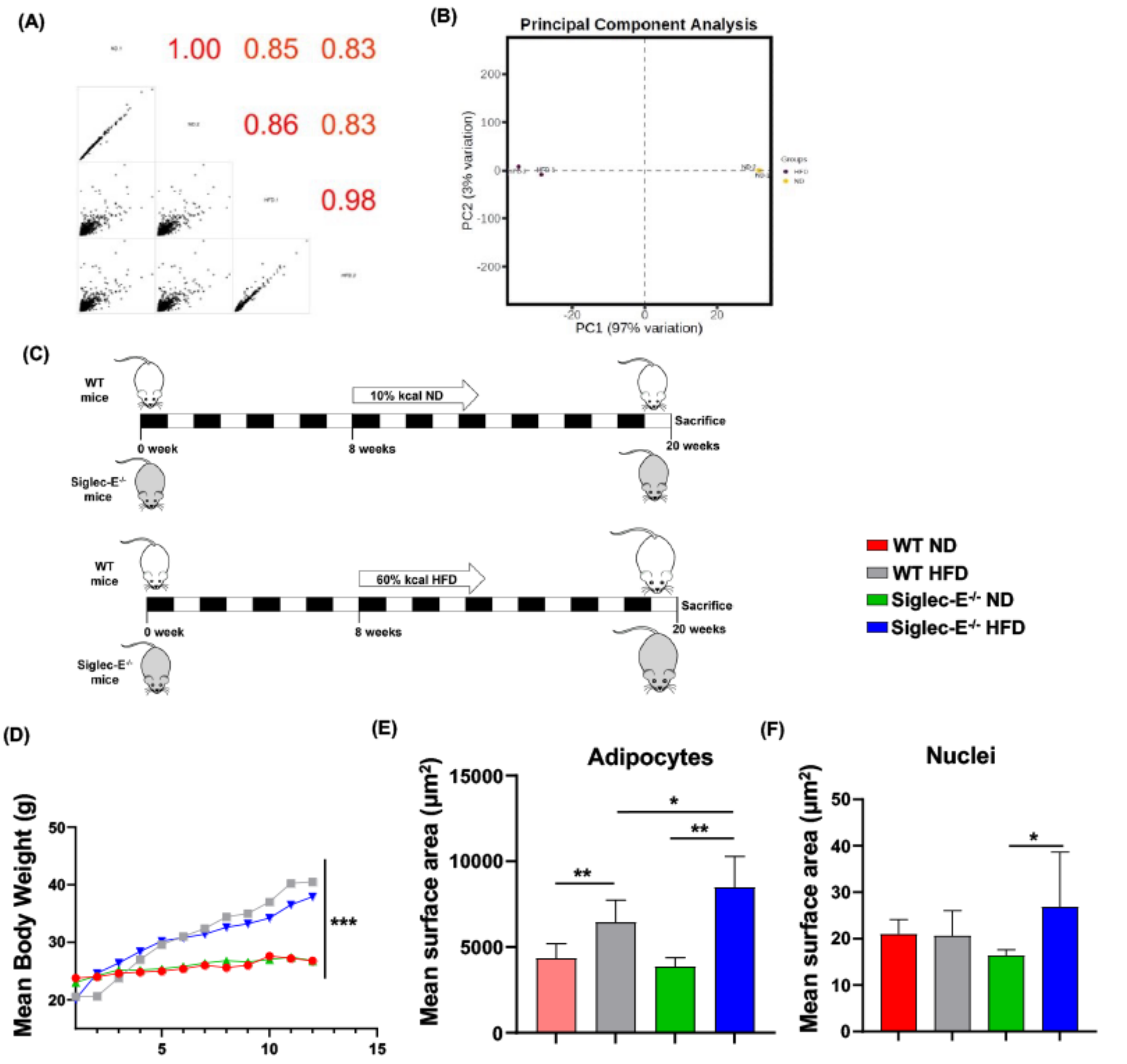

Figure S1. siglec-E is associated with adiposity during obesity condition. (A) Scatter plot for DEGs in ND-fed and HFD-fed WT animals. (B) Principal component analysis for the gene set. (C) Study design for the obesity experiment using WT and siglec-E KO mice. (D) Mean body weight of WT and Siglec-E$^{-/-}$ mice during the course of ND and HFD induction. Mean surface area of (E) adipocytes and (F) adipocytes nuclei after 12 weeks of ND and HFD feeding to both WT and siglec-E KO mice. Data are expressed as mean ± SEM; from three independent experiments (n = 5 mice per group). Statistical significance was calculated using one-way ANOVA.

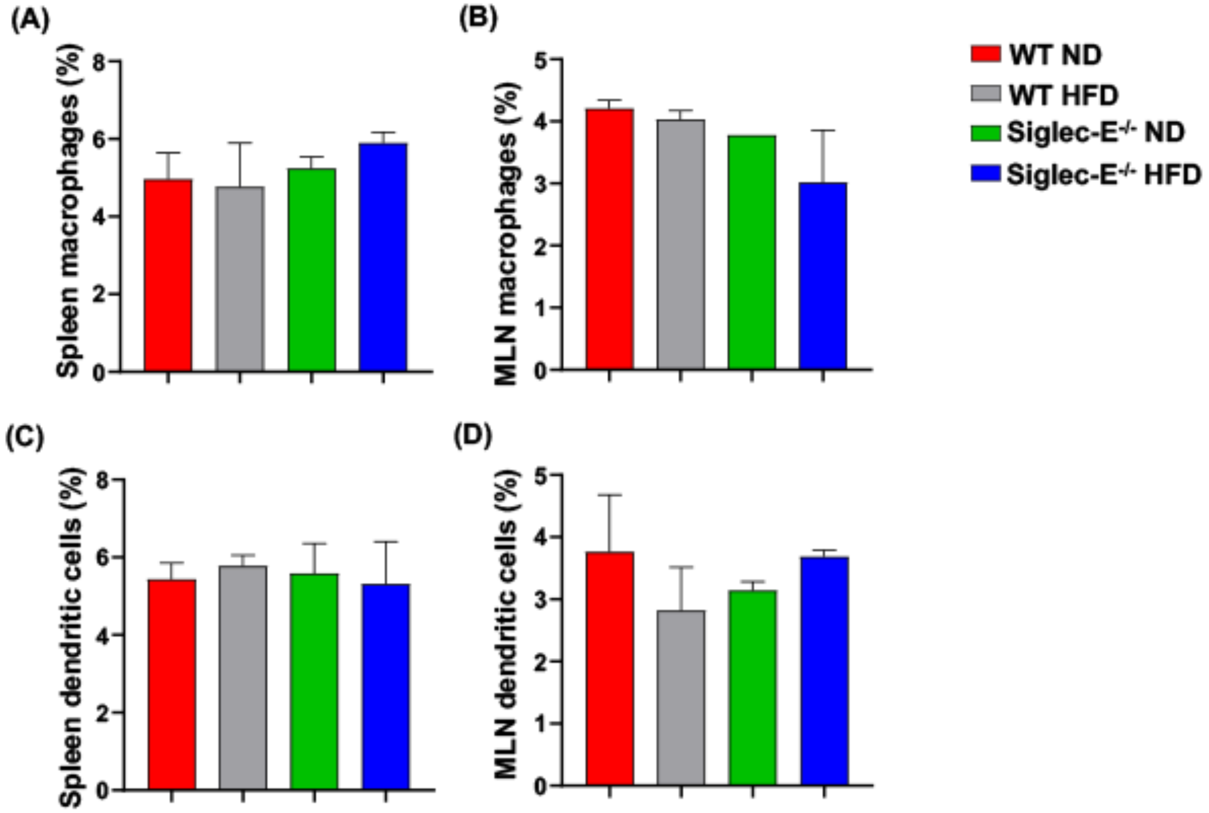

Figure S2. macrophages and DCs frequency do not significantly alter in spleen and MLNs after deletion of Siglec-E during obesity. The changes of percentages of macrophages in the (A) spleen and (B) MLN of the ND and HFD-fed WT and siglec-E KO animals. The changes of percentages of DCs in the (C) spleen and (D) MLN of the ND and HFD-fed WT and siglec-E KO animals. Data are expressed as mean ± SEM; from three independent experiments (n = 5 mice per group). Statistical significance was calculated using either one-way ANOVA or Student's T-test.

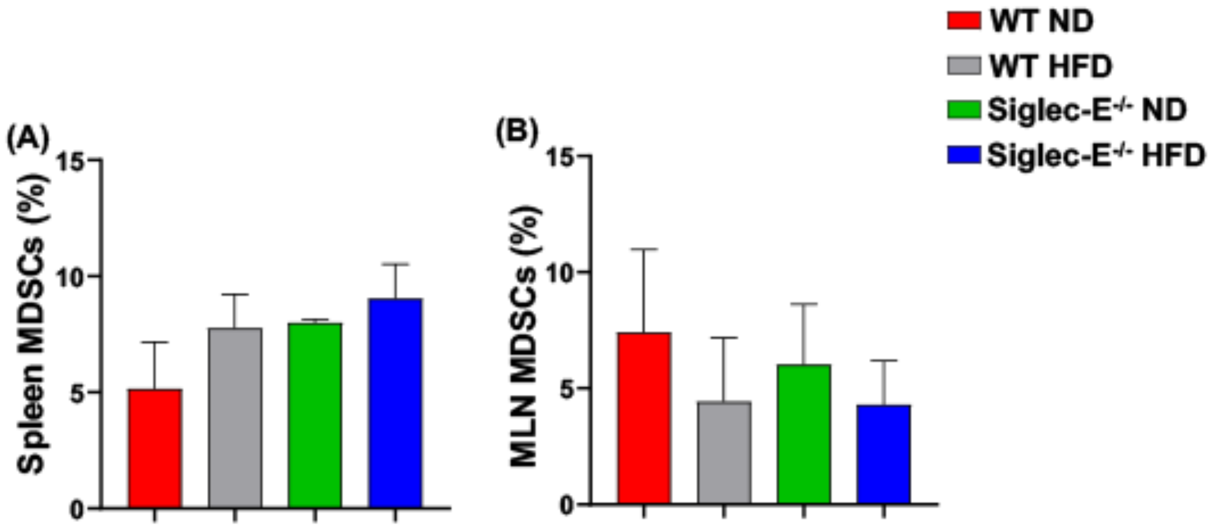

Figure S3. MDSCs frequency do not significantly alter in spleen and MLNs after deletion of Siglec-E during obesity. The changes of percentages of MDSCs in the (A) spleen and (B) MLN of the ND and HFD-fed WT and siglec-E KO animals. Data are expressed as mean ± SEM; from three independent experiments (n = 5 mice per group). Statistical significance was calculated using either one-way ANOVA or Student's T-test.

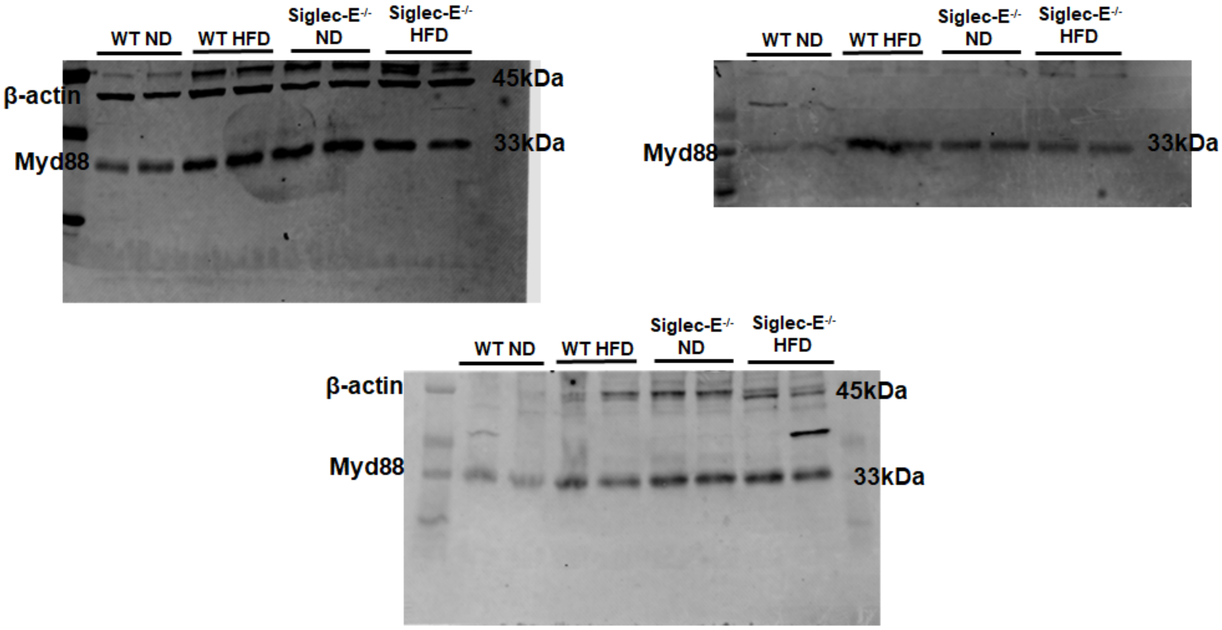

Figure S4A: Original western blot images of protein expression of MyD88

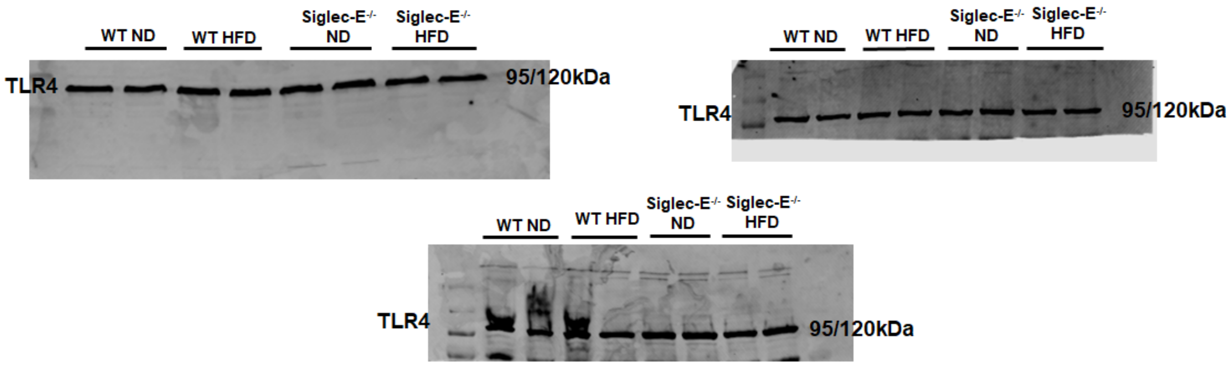

Figure S4B: Original western blot images of protein expression of TLR4

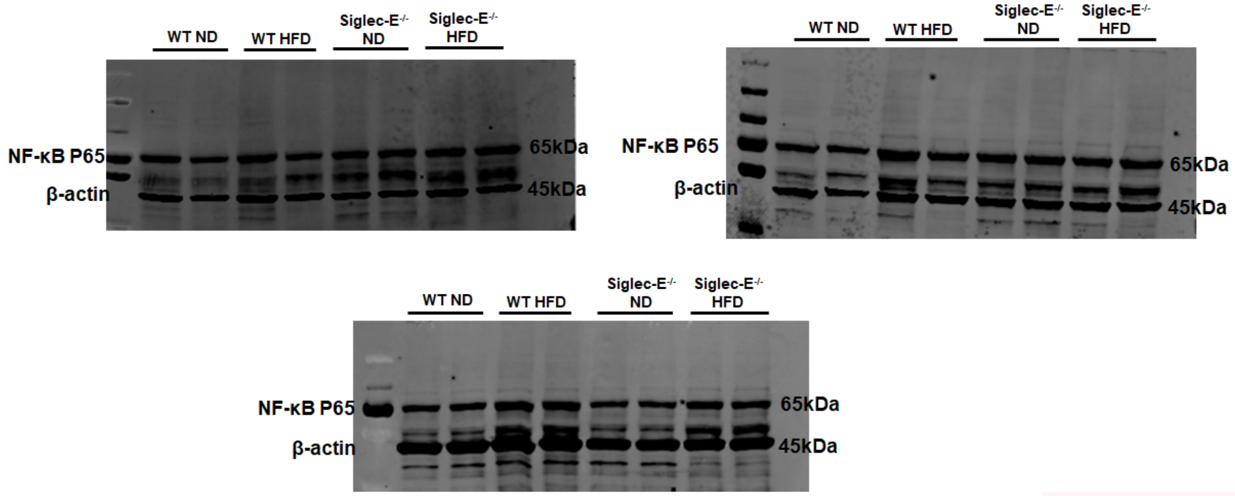

Figure S4C: Original western blot images of protein expression of NF-κB P65

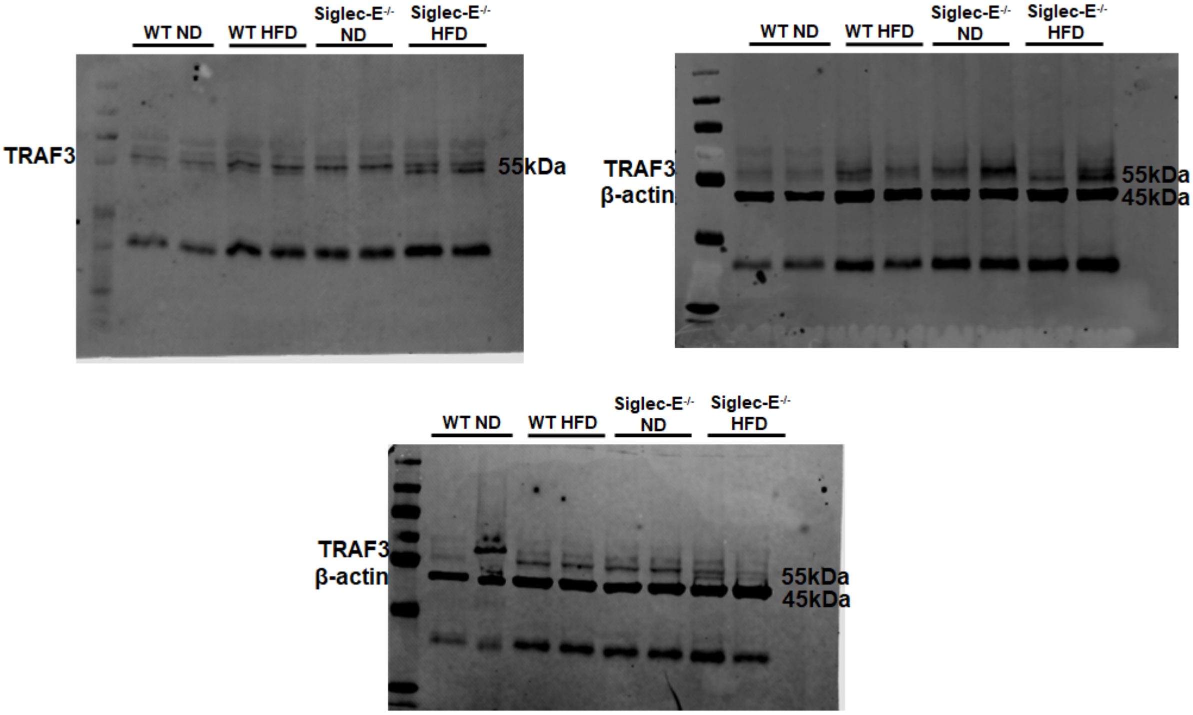

Figure S4D: Original western blot images of protein expression of TRAF3

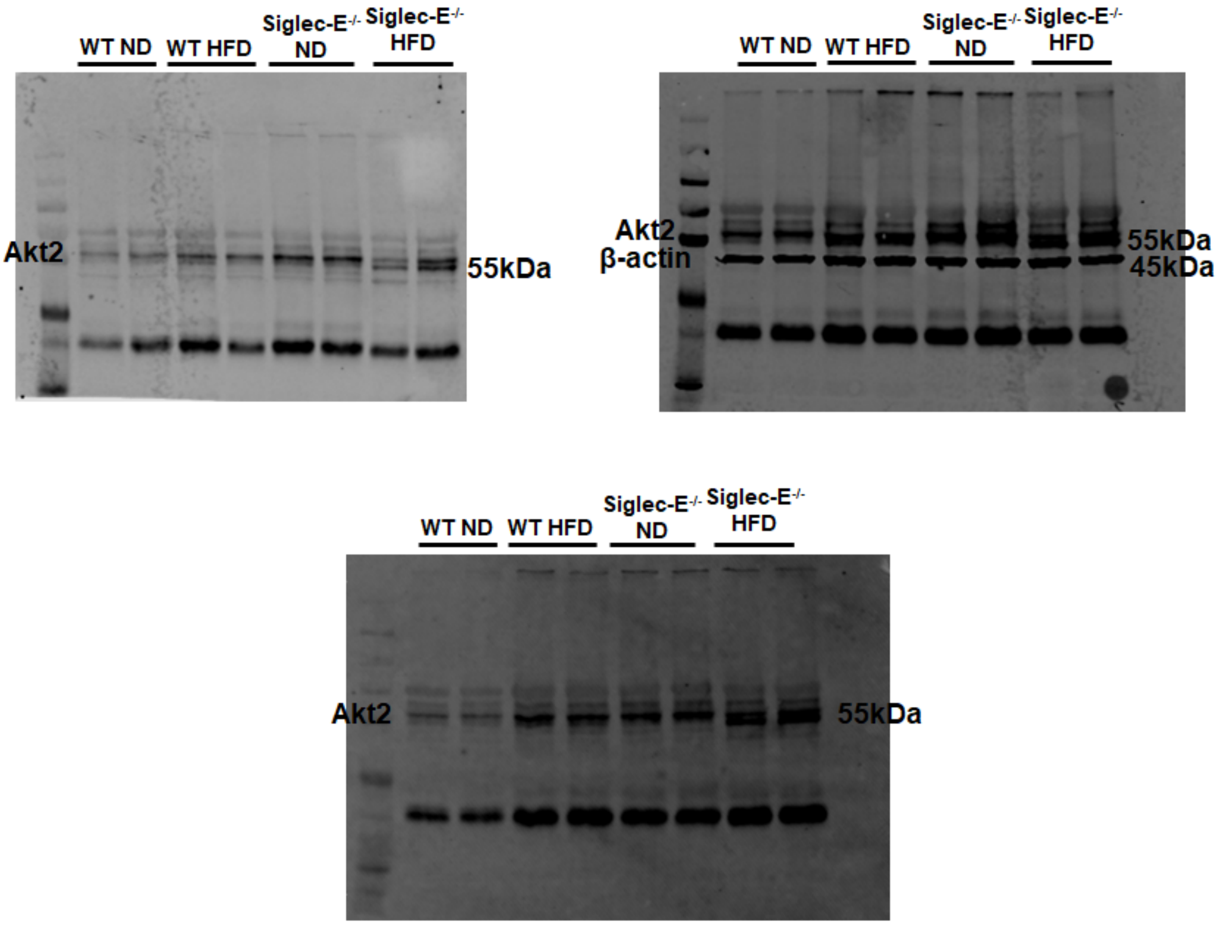

Figure S4E: Original western blot images of protein expression of Akt2

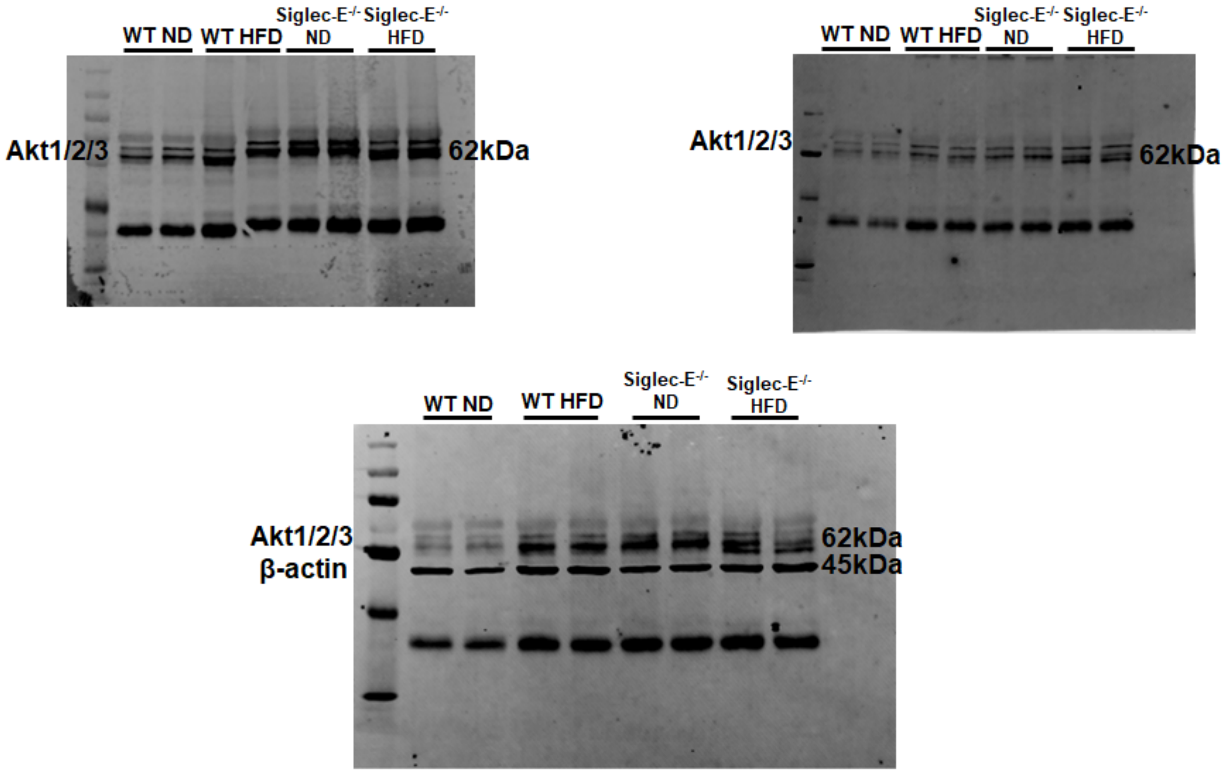

Figure S4F: Original western blot images of protein expression of Akt1/2/3

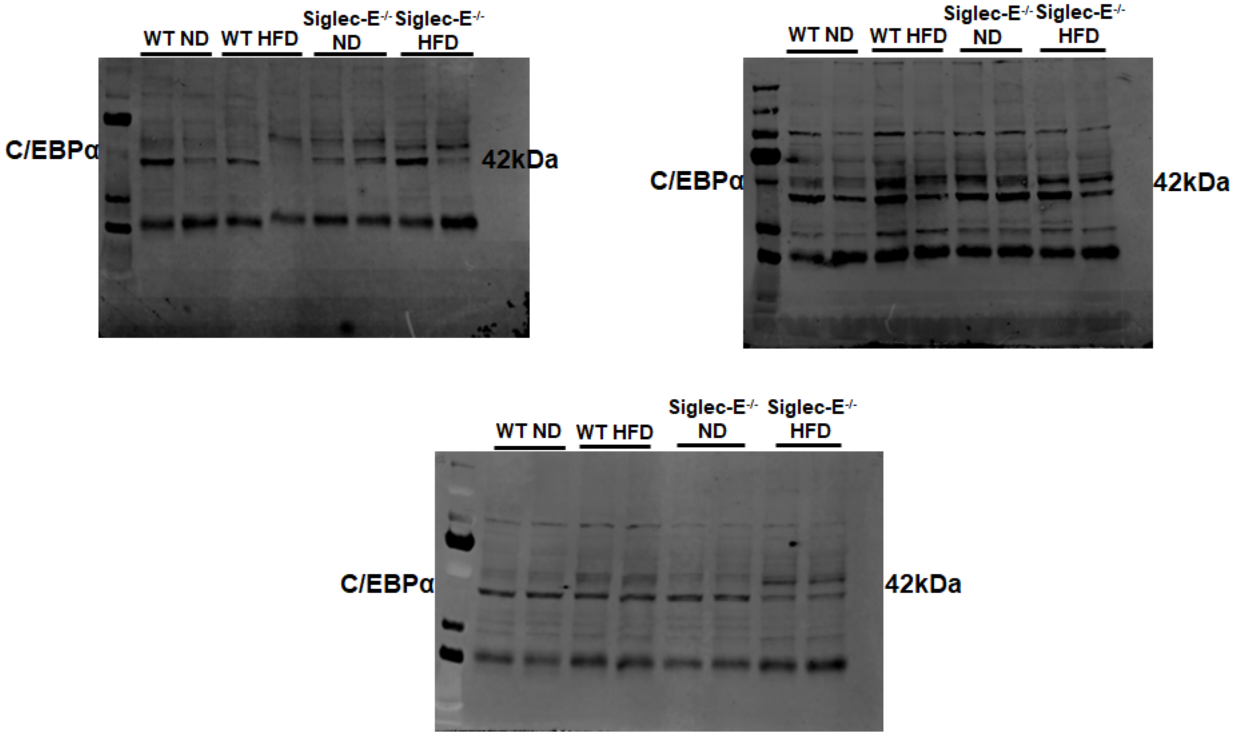

Figure S4G: Original western blot images of protein expression of C/EBPα
